# Intake of different types of seafood and meat and risk of type 2 diabetes in women: a prospective study supported by a dietary intervention in mice

**DOI:** 10.1038/s41598-024-59491-9

**Published:** 2024-04-18

**Authors:** Lene S. Myrmel, Jannike Øyen, Anne Lise Brantsæter, Even Fjære, Karen Haugvaldstad, Kåre I. Birkeland, Ottar Nygård, Karsten Kristiansen, Grace M. Egeland, Lise Madsen

**Affiliations:** 1 Institute of Marine Research, Nordnes, P.O. Box 1870, 5817 Bergen, Norway; 2 Department of Food Safety, Centre for Sustainable Diets, Norwegian Institute of Public Health, Skøyen, P.O. Box 222, 0213 Oslo, Norway; 3 Department of Transplantation Medicine, Oslo University Hospital, and Institute of Clinical Medicine, University of Oslo, Oslo, Norway; 4 Centre for Nutrition, Department of Clinical Science, University of Bergen, Bergen, Norway; 5 Mohn Nutrition Research Laboratory, University of Bergen, Bergen, Norway; 6 Department of Heart Disease, Haukeland University Hospital, Bergen, Norway; 7 Department of Biology, University of Copenhagen, Universitetsparken 13, Copenhagen Ø, Denmark; 8 Department of Health Registry Research and Development, Division of Health Data and Digitalisation, Norwegian Institute of Public Health, Sentrum, P.O. Box 973, 5808 Bergen, Norway; 9 Department of Global Public Health and Primary Care, University of Bergen, P.O. Box 7800, 5200 Bergen, Norway; 10 Department of Clinical Medicine, University of Bergen, P. O. Box 7804, 5200 Bergen, Norway

**Keywords:** Type 2 diabetes, Seafood, Shellfish, Meat, Protein intake, Protein source, Metabolism, Glucose tolerance, Metabolomics, Women, Mice, Diet, BMI, Diabetes, Risk factors, Epidemiology, Nutrition

## Abstract

Detailed knowledge regarding the associations between intake of different types of seafood and meat and the risk of type 2 diabetes (T2D), and insight into possible mechanisms are warranted. In this study we aimed to evaluate the associations between intake of different types of seafood and meat and the subsequent risk of T2D using the Norwegian Mother, Father, and Child Cohort Study (MoBa), and furthermore, by using a mouse model to gain further insight into possible molecular mechanisms contributing to the associated metabolic changes. Women in MoBa who were free of pharmacologically treated diabetes at baseline (*n* = 60,777) were prospectively evaluated for incident T2D, identified on the basis of medication usages > 90 days after delivery, ascertained by the Norwegian Prescription Database. Dietary intake was obtained with a validated 255-item food frequency questionnaire which assessed habitual diet during the first 4–5 months of pregnancy. Metabolic phenotypes and plasma metabolome were investigated in female mice fed isocaloric diets with different types of seafood and meat mimicking the dietary intake in the human cohort. During maximum 10-year and mean (SD) 7.2 (1.6) years follow-up time, 681 (1.1%) women developed pharmacologically treated T2D. All statistical models identified a higher risk of T2D with increased shellfish intake, whereas no associations were observed for total seafood, fatty fish, total meat and red meat in the adjusted models. In mice, the shellfish-based western diet induced reduced glucose tolerance and insulin secretion compared to the diet based on lean fish, and we identified a number of metabolites elevated in plasma from shellfish-fed mice that correlated with glucose intolerance. Mice fed a western diet based on meat also exhibited reduced glucose tolerance in comparison to lean fish fed mice, whereas mice fed fatty fish, total seafood or red meat did not differ from lean fish fed mice. We observed a diet-specific metabolic signature in plasma demonstrating five distinct metabolite profiles in mice fed shellfish, fatty fish, total seafood/lean fish, a mixed diet and meat. In conclusion, these findings demonstrate that different types of seafood have different outcome on T2D risk. In women, intake of shellfish was associated with higher risk of T2D. In female mice, a shellfish enriched diet reduced glucose tolerance and altered the abundance of several distinct plasma metabolites correlating with glucose tolerance.

## Introduction

Eating a healthy diet, regular physical activity, and maintaining normal weight are important tools to prevent the development of insulin resistance and type 2 diabetes (T2D). With regard to the diet, meta-analyses of cohort studies have provided evidence that intake of meat and red meat in particular is associated with increased risk of T2D, whereas studies investigating the association between intake of seafood and risk for T2D are inconclusive^1–4^. Moreover, the association between meat intake and T2D risk is found to be attenuated when adjusting for BMI in prospective cohort studies^3^. Of note, a recent meta-analysis of randomized controlled trials suggests no impact from red meat intake on risk factors for T2D^5^ implicating the need for further in-depth studies. The discrepancy between different studies concerning intake of fish and other seafood may relate to the amount and type of seafood in the diet or cultural eating habits and preparation methods^6^. Some studies indicate that lean, but not fatty fish may protect against T2D^7,8^, whereas intake of shellfish has been associated with an increased risk of T2D in some^9–11^ but not all studies^12–15^. The impact of seafood intake on the risk of T2D is found to be influenced by the prevalence of obesity in the population. In populations with high prevalence of obesity, a high fish and seafood intake may reduce the risk of T2D, whereas no association between T2D and fish and seafood intake was found in populations with a low rate of obesity^16^.

More knowledge regarding the associations between intake of different types of seafood and meat and the risk of T2D, and insight into possible mechanisms are warranted. Therefore, the aim of this study was to evaluate the associations between different types of seafood and meat and subsequent risk of T2D in women in the Norwegian Mother, Father, and Child Cohort Study (MoBa). To gain insight into metabolic changes that may influence the development of T2D and provide information on possible causal links between the intake of different dietary protein sources and the risk of T2D, we fed female mice seven different isocaloric diets mimicking the average dietary intake of the participants in MoBa, enriched with total seafood, lean fish, fatty fish, shellfish, total meat, or red meat and followed changes in metabolic phenotypes and the plasma metabolome.

## Material and methods

### Population and design

MoBa is a prospective nation-wide population-based pregnancy cohort study conducted by the Norwegian Institute of Public Health. Pregnant women from all regions in Norway were recruited to participate from 1999 to 2008 by postal invitation prior to their first scheduled ultrasound around 18 weeks of gestation. The women consented to participation in 41% of the pregnancies. The cohort now includes around 114,500 children, 95,200 mothers and 75,200 fathers, while in the current study, only data from the mothers were included. Follow-up was conducted through questionnaires and by linkage to national health registries^17^.

MoBa was linked to the Medical Birth Registry of Norway (MBRN)^18^ for information regarding pregnancy-related risk factors, the Norwegian Prescription Database (NorPD) for information about use of diabetes medication, and the National Population Registry for information about migration and deaths. MBRN is a compulsory registry containing information for all births (> 16 weeks gestation) in Norway and the NorPD is a registry containing information on all dispensed prescriptions to non-institutionalized individuals established in 2004. To be eligible for inclusion in the current study, women had to be registered in MBRN and delivered a baby between 2004 and 2009 (last pregnancy for those who participated in MoBa more than once), had completed the baseline questionnaire and the dietary questionnaire in pregnancy, and still participate in MoBa (*n* = 62,547 women). We excluded women with invalid energy intakes (< 1070 or > 4400 kcal/d) (*n* = 1351)^19^, with diabetes prior to pregnancy ascertained by three data sources (MoBa, MBRN, and NorPD) (*n* = 439), and nonviable births (birth weight < 500 g or when missing birth weight at gestational age < 22 weeks) (*n* = 15). With minor overlap in exclusions, a total of 60,777 women remained eligible for the analyses (Fig. S1).

### Dietary intake

Information on dietary intake was obtained using a validated 255-item semi-quantitative food frequency questionnaire (FFQ) answered around week 22 of pregnancy^20^. The MoBa FFQ was validated in 119 MoBa participants using a four-day weighed food diary, motion sensors as a marker of energy expenditure, and biological markers measured in blood and 24-h urine samples as reference methods^20,21^. The results showed that the FFQ enables reasonable ranking of the participants’ diet according to major food groups and nutrients as well as nutrient intakes through dietary supplements^20,22^.

The FFQ included 16 questions about fish or shellfish (crustaceans) eaten as dinner, 10 questions about cold cuts and spreads from fish or shellfish. Fish items (g/d) were grouped as lean fish species (i.e. 0.3–6.0% fat) and fatty fish species (i.e. 10–24% fat) and included items consumed as dinner and as cold cuts and bread spreads. Total seafood included lean and fatty fish (salt and freshwater fish, fish-based spread), liver, roe, and shellfish. The FFQ included 33 questions about meat eaten as dinner and six questions about cold cuts and spreads. Meat items (g/d) were grouped as red meat (beef, lamb and pork) and other meat (game and poultry). When estimating the intakes of fish and meat, only the fish and meat components of mixed dishes were included.

Other nutrients from the FFQ included daily energy intake (kcal), fibre, total protein, total carbohydrate, and total fat, saturated fat, monounsaturated fat, and polyunsaturated fat. FoodCalc and the Norwegian food composition table were used for nutrient calculations^20^.

### Covariates

The baseline pre-pregnancy covariates came from the first MoBa questionnaire administered at 15–17 weeks gestation and included daily pre-pregnancy cigarette smoking, height and weight for calculating BMI, educational level, marital status, and leisure-time physical activity.

The MBRN provided information on maternal age at delivery, parity, chronic illness and pregnancy-related complications (gestational or unspecified diabetes, gestational hypertension/preeclampsia), and whether pregnancy was a multiple birth pregnancy.

Gestational diabetes mellitus was ascertained through three sources: MoBa questionnaire, MBRN, and the use of medications (insulin or metformin) noted in the NorPD during pregnancy.

### Ascertainment of type 2 diabetes

T2D was ascertained by identifying all women who initiated treatment with one or more anti-hyperglycemic agents (Anatomical Therapeutic Chemical classification code, ATC A10) with a dispensed prescription of at least 90 days or more following delivery. This included both insulin and oral agents. To exclude subjects with type 1 diabetes, we excluded users of insulin-only to the end of follow-up*.* A total of 709 ATC A10 medication users were identified of whom we excluded 28 presumed to have type 1 diabetes.

### Statistical analyses of data from the MoBa cohort

Descriptive characteristics and dietary intake are presented as mean and standard deviation (SD), median (5th, 95th percentile (P5, P95)) or percentages for continuous and categorical variables, respectively. Descriptive characteristics and dietary intake are also presented by quintiles of shellfish intake. All dietary intakes were energy-adjusted using the nutrient density method^23^, expressing foods as g/1000 kcal or E%.

Cox proportional hazard analyses were used to evaluate the association between both protein intake and the whole sources of seafood and meat, and relevant sub-categories, and development of T2D up to 10 years following delivery (i.e., through December 31, 2014). All intakes were evaluated both as continuous variables and as ranked into quintiles. Due to a high percentage of non-consumers of shellfish (34.8%), we defined shellfish non-consumers as the lowest quantile and ranked consumers into quartiles and present these five groups as quintiles. The HRs for relevant protein sources are reported per g/1000 kcal in daily intake, and the HRs for the whole sources are shown per 25 g/1000 kcal or 5 g/1000 kcal (for shellfish) increment in daily intake.

We adjusted the Cox regression models for potential confounders using three models: (1) energy intake and age, (2) model 1 plus BMI (kg/m^2^), gestational diabetes, gestational hypertension/preeclampsia, and (3) model 2 plus maternal education, pre-pregnancy smoking, and dietary fibre. Dietary fibre was chosen, *a-priori*, as a proxy of overall dietary quality as it reflects the intake of vegetables, whole grain and a dietary pattern in line with healthy eating. Additional adjustments for marital status and physical activity were evaluated together with model 3 covariates, but as their inclusion did not alter the results they were not included in the final models presented (results not shown).

To study the dose–response associations between protein intake from seafood and meat (and the relevant sub-categories) and the risk of T2D, we computed Cox proportional hazard regression model plots with model 3 adjustments.

We also explored consistencies in results in analyses stratified by BMI categories (< 25 and ≥ 25 kg/m^2^).

The proportionality assumptions for the Cox models were evaluated graphically by log-minus-log-plots. The HRs were stable, and for all models it did not violate the proportionality assumption.

There were 1435 (2.3%), 1185 (1.9%), and 303 (0.5%) women with missing baseline values for pre-pregnancy BMI, maternal education, and civil status, respectively. Missing values were imputed using multiple imputation for Statistical Package for the Social Science (SPSS). Two-tailed *P* values < 0.05 were considered statistically significant. The analyses were performed using IBM SPSS Statistics for Windows, version 29 (IBM Corp. Armonk, NY, USA). The Cox proportional hazard regression plots were generated using R (R Core Team, 2020) operated in RStudio (version 1.3.959; RStudio Team, 2015).

### Experimental animal design

Eighty female C57BL/6J mice, 8 weeks old, were obtained from Charles River (Germany) and individually housed in ventilated cages (IVC) at thermoneutral conditions (29 ± 1 °C) with 50% relative humidity and a 12:12 h light–dark cycle.

Prior to experimental start, the mice were acclimated for one week and provided a low-fat reference diet (AIN-93G, Growth Purified Diet, TestDiet^®^) ad libitum. During the entire experiment, all mice were fed ad libitum, given new feed three times per week, fresh water provided once per week and all feed remnants recorded every second week. Body weight development was monitored by weighing once a week. Body composition was measured at start to ensure similar group means of body weight, lean mass and fat mass in all experimental groups. All mice were distributed into eight experimental groups (*n* = 10) given the experimental diets (Table S1). All mice were randomized to diet regimen to balance body composition among all experimental groups at start. All procedures and samples were taken and prepared in a randomized order, distributing mice and samples from the different experimental groups according to variation in time and location. The animal caretaker was aware of the group allocation due to the different experimental feeding, while further analyses of samples and data were blinded when possible. The number of animals was calculated based on data from a previous animal trial using similar diets and the same mouse strain.

As a primary outcome an oral glucose tolerance test (GTT) was performed in week 11 of the feeding experiment. After 5-h feed deprivation, an oral dose of 3 mg glucose/g lean body mass was given by gavage. Blood samples were collected from the lateral tail vein by a small cut in the tail tip. Blood glucose levels were measured at baseline, and 15, 30, 60, and 120 min post oral glucose load. At baseline and after 30 and 120 min, whole blood samples were collected in EDTA anticoagulant tubes and plasma centrifuged at 2500 × *g* for 10 min, at 4 °C, to obtain plasma for insulin and glucose measurements. Blood glucose was measured using a glucometer (Contour, Bayer) and plasma insulin using an Ultra-Sensitive Mouse Insulin ELISA Kit (Chrystal Chem Inc., USA). The incremental area under the curve (iAUC) (y = baseline (blood glucose level at time 0)) of the glucose levels during GTT was determined by GraphPad Prism 9.

At termination of the experiment, the mice were anesthetized with 4% isoflurane (Isoba-vet, Schering-Plough A/S, Farum, Denmark) using a Univentor 400 Anesthesia Unit Apparatus and blood collected in EDTA anticoagulant tubes by cardiac puncture. Plasma samples were prepared by centrifugation at 2500 × *g* at 4 °C for five minutes.

### Mouse diets

The seven experimental diets given to mice were based on the average macronutrient intake data from the women in MoBa, using different protein sources (Table S1). All essential micronutrients were included based on the 5TJN standard western diet for rodents (TestDiet). The protein sources comprised a mixture of proteins from total seafood, lean fish, fatty fish, shellfish, total meat, and red meat as described in Tables S2. As a reference we prepared a diet with a mixture of protein sources based on food intake data registered in the MoBa study. In addition, a standard low-fat reference diet (AIN-93G, Growth Purified Diet, TestDiet^®^) was used as a second reference. All protein sources were cooked to a core temperature of 75 °C using a steamer and frozen at − 20 °C. Protein sources within each category (Table S2) were thoroughly mixed, and equal amounts of protein from each category were added to the diets in amounts calculated from measurements of nitrogen content, equal to 160 g crude protein/kg (Table S1). Nitrogen was determined by the Dumas method using a Leco FP 628 nitrogen analyzer (Leco Corporation Svenska AB, Sweden), using Nx5.6 as the nitrogen-to-protein conversion factor^24^. The endogenous total fat content in the protein powders was determined gravimetrically after extraction with organic solvents before and after acidic hydrolysis as described by Tastesen et al.^25^. To obtain an equal amount of 160 g total fat/kg diet in all experimental diets, vegetable shortening, milk fat, lard, soybean oil and corn oil were included (Table S1). The diets were blended using a Crypto Peerless EF20 blender and analysed for gross energy by bomb calorimetry (Parr Instrument, Moline, IL, USA). The levels of amino acids (Table S3) and fatty acids (Table S4) were measured in all experimental diets as earlier described^25^.

### Metabolomic analysis

Metabolomic analysis of mouse plasma samples was performed by Metabolon’s global biochemical profiling (HD4) and complex lipid panel (CLP) platforms^26^. Plasma samples stored at − 80 °C from eight mice per experimental group were extracted and analysed on the LC/MS/MS and polar LC platform at Metabolon. Due to the high cost of metabolomic analyses, only eight plasma samples per experimental group were analysed. Further, ions were matched to their in-house library of standards for metabolite identification and quantification by peak area integration, all performed by Metabolon as described earlier^26^. Missing values were imputed with the observed minimum for that particular compound for all analyses. A total of 760 and 926 biochemicals were detected by the global (HD4) and CLP platforms at Metabolon, respectively. The plasma samples for metabolomic analyses were chosen to keep the same mean of the AUC for the GTT and body weight in each experimental group as the mean when all ten samples per group were present.

### Statistical analysis of animal experiment data

Data from the experimental animal groups presented in graphs demonstrate mean ± standard error of the mean (SEM). Outliers were identified using Grubbs’ method and removed. Brown-Forsythe test was performed to determine the homogeneity of variance. ANOVA with uncorrected Fisher’s Least Significant Difference (LSD) for multiple comparisons was performed to compare the experimental groups if homogeneity of variance was detected. Kruskall-Wallis test was performed as indicated when variances were significantly different. All graphs and included statistics were performed by GraphPad Prism 9 (GraphPad Software Inc). Principal component analysis (PCA) plots of metabolites, heat maps with hierarchical clustering analysis of all metabolites exhibiting significant changes in abundance as determined by ANOVA (q < 0.05) and rank regression to determine metabolites significantly correlated with glucose tolerance were generated by the use of Qlucore omics explorer (2018 Qlucore AB).

### Ethical approval

This study was carried out in accordance with relevant guidelines and regulations and all protocols were approved by the relevant agencies and committees, including Regional Committees for Medical and Health Research Ethics, the Norwegian Animal Research Authority and the European Convention for the Protection of Animals used for scientific purposes. An informed consent was obtained from all participants. The establishment of MoBa and initial data collection were based on a license from the Norwegian Data Protection Agency and approval from the Regional Committees for Medical and Health Research Ethics. The MoBa cohort is currently regulated by the Norwegian Health Registry Act. The current study is an extension of a research project evaluating risk factors for chronic hypertension development within 10 years following delivery^27^, approved by all relevant agencies and the Regional Committees for Medical and Health Research Ethics (Region West 2013/740) with amendments (14.03.2019) for including T2D as an outcome measure. This study is based on version 12 of the quality-assured data files released for research in 2019.

The animal experiment was approved by the Norwegian Animal Research Authority (FOTS ID nr. 19477). Handling of mice and conduction of experimental protocols were in accordance with the guidelines from the national authorities and the European Convention for the Protection of Animals used for scientific purposes. The study is reported in accordance with the ARRIVE guidelines.

### Prior presentation of data

Some of the data from MoBa have been published earlier^8^.

## Results

### Participant characteristics and dietary intakes

Descriptive characteristics of the total cohort and by quintiles of shellfish intake are presented in Table 1 and Table S5, respectively. The participants mean (SD) age at time of delivery was 30.5 (4.6) years, pre-pregnancy weight was 68.1 (12.8) kg, and BMI was 24.0 (4.2) kg/m^2^ with 68.3% having a BMI < 25 kg/m^2^. Before pregnancy, 97.0% were married or had a partner, 15.9% smoked daily, 48.0% engaged in leisure-time physical activity ≥ 3 times per week, and 28.2% had an education level of ≥ 17 years (Table 1).

**Table 1:** Baseline characteristics and the energy-adjusted daily dietary intake (*n* = 60,777).

	Total cohort	
	Mean (SD)	Median (P5, P95)
Age at delivery, years	30.5 (4.6)	31.0 (23.0, 38.0)
Height, cm	168 (0.06)	168 (158, 178)
Pre-pregnancy variables		
Weight, kg	68.1 (12.8)	65.0 (52.0, 93.0)
BMI, kg/m^2^	24.0 (4.2)	23.1 (18.9, 32.5)
BMI < 25 kg/m^2^ (%)	68.3	–
Married/partner (%)	97.0	–
Daily smoking (%)	15.9	–
Physical activity ≥ 3 times/wk (%)	48.0	–
Education ≥ 17 years (%)	28.2	–
Pregnancy variables		–
Gestational diabetes (%)	1.0	–
Gestational hypertension (%)	2.1	–
Preeclampsia (%)	3.7	–
Daily intake		
Energy, kcal	2291 (592)	2216 (1446, 3416)
Total seafood*, g/1000 kcal	16.4 (10.0)	14.8 (2.9, 34.8)
Lean fish, g/1000 kcal	9.2 (6.6)	8.0 (0.00, 21.2)
Fatty fish, g/1000 kcal	4.9 (5.6)	3.3 (0.00, 15.3)
Shellfish, g/1000 kcal	1.6 (2.2)	1.0 (0.00, 5.4)
Total meat^†^, g/1000 kcal	70.3 (24.0)	68.0 (35.9, 113)
Red meat, g/1000 kcal	57.7 (22.4)	55.4 (25.3, 97.7)
Other meat^‡^, g/1000 kcal	12.7 (8.9)	10.7 (1.5, 29.3)
Milk/dairy, g/1000 kcal	220 (135)	204 (43.7, 470)
Bread/cereals/pasta, g/1000 kcal	134 (36.4)	133 (75.8, 195)
Eggs, g/1000 kcal	11.1 (7.6)	9.0 (3.0, 25.3)
Vegetables/fruits/nuts, g/1000 kcal	359 (159)	336 (145, 646)
Cholesterol, mg/1000 kcal	108 (28.4)	103 (72.9, 157)
Fibre, g/1000 kcal	13.5 (3.1)	13.3 (9.0, 19.0)
Added sugar, E%	10.3 (4.7)	9.5 (4.1, 18.7)
Protein, E%	15.5 (2.1)	15.4 (12.2, 19.0)
Carbohydrate, E%	53.5 (4.7)	53.4 (46.0, 61.1)
Total fat, E%	30.7 (4.5)	30.6 (23.5, 38.0)
Saturated fat, E%	11.8 (2.1)	11.7 (8.6, 15.3)
Monounsaturated fat, E%	9.9 (1.8)	9.7 (7.1, 12.9)
Polyunsaturated fat, E%	5.8 (1.5)	5.4 (3.7, 8.6)

Data are mean (SD), median (5th, 95th percentile (P5, P95)) or percentage.

*Total seafood includes lean and fatty fish (salt and freshwater fish, fish-based spread), liver, roe, and shellfish.

^**†**^Total meat includes red meat (beef, pork, mutton, processed red meat), game, poultry.

^‡^Other meat includes game, poultry.

The energy-adjusted dietary intakes in the total cohort and by quintiles of shellfish intake are presented in Table 1 and Table S5, respectively. The energy-adjusted intake of protein from different sources is presented in Table S6. The participants had a mean (SD) daily energy-adjusted intake of total seafood, lean fish, fatty fish, and shellfish of 16.4 (10.0), 9.2 (6.6), 4.9 (5.6), and 1.6 (2.2) g/1000 kcal, respectively. The intake of total meat, red meat and other meat was 70.3 (24.0), 57.7 (22.4), and 12.7 (8.9) g/1000 kcal/d, respectively (Table 1). Mean (SD) absolute intake of total seafood, lean fish, fatty fish, shellfish, total meat, red meat, and other meat was 36.0 (21.6), 19.9 (13.1), 11.0 (13.0), 3.5 (5.1), 152 (39.2), 125 (40.5), and 27.1 (16.7) g/day, respectively. The percentage of non-consumers of shellfish was 34.8%. For total seafood, lean fish, fatty fish, total meat, red meat, and other meat, 2.2%, 5.3%, 12.7%, 0.10%, 0.10%, and 3.7% were non-consumers, respectively.

### Associations between seafood and meat intakes and type 2 diabetes

During the maximum 10-year and mean (SD) follow-up time of 7.2 (1.6) years, 681 (1.1%) participants developed pharmacologically treated T2D, of which 191 had a diagnosis of gestational diabetes during the index pregnancy. In Cox regression analyses, all models identified a higher risk of T2D with increased energy-adjusted shellfish intake (model 3: HR 1.20, 95% CI 1.06–1.36, *P* = 0.004) per 5 g shellfish/1000 kcal. In contrast, and as presented previously^8^, a lower risk of T2D associated with intake of lean fish (Table 2). For intake of total meat and red meat, a higher risk of T2D was found in model 1, but not after adjusting for BMI and other covariates. No associations were observed for intakes of total seafood, fatty fish, and other meat (Table 2). Similar findings were observed when we evaluated the energy-adjusted protein intake from seafood and meat (Table S7). We observed an inverse association between protein intake from lean fish and T2D, whereas a positive relationship was observed between intake of shellfish and T2D (Fig. S2).

**Table 2:** HRs (95% CIs) for incident type 2 diabetes (T2D) by energy-adjusted seafood and meat intake in 60,777 women with 681 T2D events.

	Model 1*	Model 2^†^	Model 3^‡^
Total seafood^§^ (25 g/1000 kcal)	0.86 (0.71–1.05);	0.92 (0.76–1.11);	0.91 (0.75–1.10);
	*P* = 0.134	*P* = 0.362	*P* = 0.315
Lean fish (25 g/1000 kcal)	0.67 (0.49–0.91);	0.72 (0.54–0.97);	0.70 (0.52–0.94);
	*P* = 0.011	*P* = 0.029	*P* = 0.018
Fatty fish (25 g/1000 kcal)	0.83 (0.58–1.19);	0.93 (0.66–1.31);	0.94 (0.67–1.32);
	*P* = 0.309	*P* = 0.673	*P* = 0.732
Shellfish (5 g/1000 kcal)	1.18 (1.04–1.34);	1.19 (1.05–1.35);	1.20 (1.06–1.36);
	*P* = 0.008	*P* = 0.005	*P* = 0.004
Total meat^||^ (25 g/1000 kcal)	1.22 (1.11–1.35);	0.99 (0.90–1.09);	0.99 (0.89–1.09);
	*P* < 0.001	*P* = 0.820	*P* = 0.786
Red meat (25 g/1000 kcal)	1.22 (1.11–1.34);	0.98 (0.89–1.08);	0.98 (0.89–1.08);
	*P* < 0.001	*P* = 0.740	*P* = 0.669
Other meat^¶^ (25 g/1000 kcal)	0.97 (0.77–1.22);	1.03 (0.83–1.28);	1.04 (0.84–1.30);
	*P* = 0.794	*P* = 0.804	*P* = 0.718

Data are HR (95% CI) unless otherwise indicated.

*Adjusted for energy intake and age.

^†^Adjusted for energy intake, age, pre-pregnancy BMI, gestational diabetes, and gestational hypertension including preeclampsia.

^‡^Adjusted for energy intake, age, pre-pregnancy BMI, gestational diabetes, gestational hypertension including preeclampsia, maternal education, pre-pregnancy smoking, and dietary fibre.

^§^Total seafood includes lean and fatty fish (salt and freshwater fish, fish-based spread), liver, roe, and shellfish.

^||^Total meat includes red meat (beef, pork, mutton, processed red meat), game, and poultry.

^¶^Other meat includes game and poultry. The total seafood, lean fish and fatty fish results have been published previously (10).

**Figure 1:**
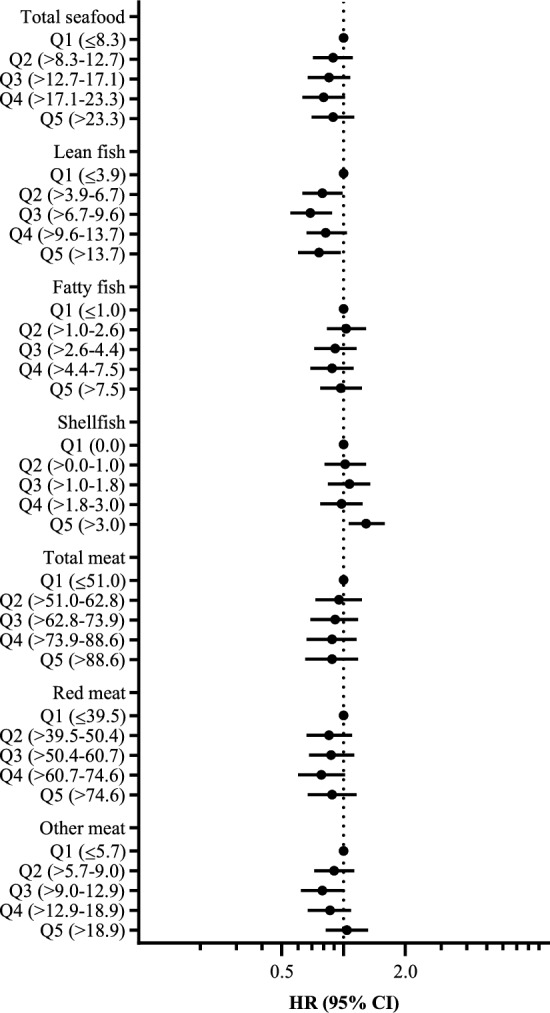
Forest plot showing the associations (HRs and 95% CIs) between quintiles of energy-adjusted seafood and meat intake (g/1000 kcal) and incident type 2 diabetes (T2D) in 60,777 women with 681 T2D events. Adjusted for model 3: energy intake, age, pre-pregnancy BMI, gestational diabetes, gestational hypertension including preeclampsia, maternal education, pre-pregnancy smoking and dietary fibre. Total seafood includes lean and fatty fish (salt and freshwater fish, fish-based spread), liver, roe and shellfish. Total meat includes red meat (beef, pork, mutton, processed red meat), game and poultry. Other meat includes game and poultry. The total seafood, lean fish and fatty fish results have been published previously^8^.

In Cox regression analyses of quintiles of energy-adjusted fish and meat intake, a higher risk of T2D was observed for shellfish intake quintile five compared to quintile one (non-consumers) (model 3: HR 1.29, 95% CI 1.05–1.59) (Fig. 1). For energy-adjusted intake of lean fish, a lower risk of T2D was found as previously published^8^. No associations were seen for total seafood, fatty fish and meat in model 3 (Fig. 1). Similar findings were observed in Cox models with use of energy-adjusted protein intakes (Fig. S3).

### Sensitivity analyses

In Cox regression analyses stratified by BMI categories (< 25 and ≥ 25 kg/m^2^), a higher risk of T2D was observed with increased energy-adjusted shellfish intake (g/1000 kcal) in both strata, indicating no interaction by BMI. In the BMI group < 25 kg/m^2^ the association between shellfish intake and T2D was statistically significant (Table S8). As previously reported, a lower risk of T2D was observed with increased intake of lean fish in the BMI group ≥ 25 kg/m^2^^8^. No associations were observed for energy-adjusted intakes of total seafood, fatty fish, red meat, and other meat (Table S8). Similar findings were observed when the energy-adjusted protein intake from seafood and meat were evaluated (Table S9).

**Figure 2:**
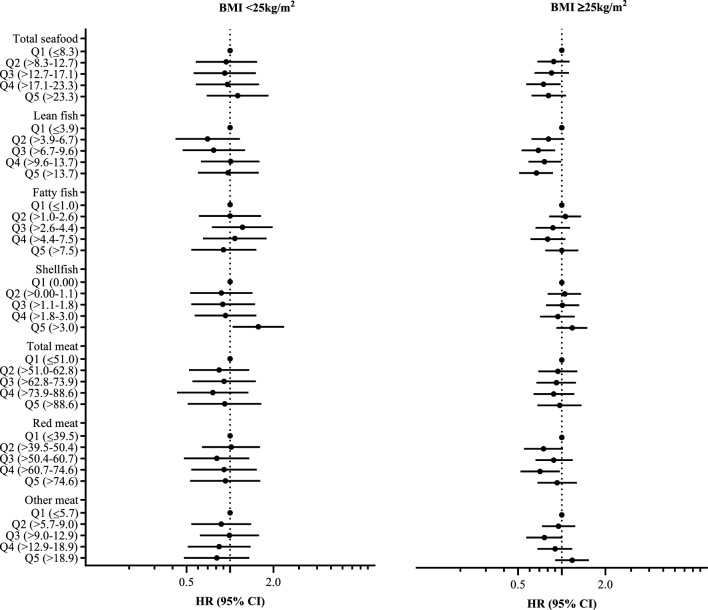
Forest plot showing the associations (HRs and 95% CIs) between quintiles of energy-adjusted intake of seafood and meat (g/1000 kcal) and incident type 2 diabetes (T2D) stratified on pre-pregnancy BMI < 25 vs ≥ 25 kg/m^2^ in 60,777 women with 681 T2D events. Adjusted for model 3: energy intake, age, gestational diabetes, gestational hypertension including preeclampsia, maternal education, pre-pregnancy smoking, and dietary fibre. Total seafood includes lean and fatty fish (salt and freshwater fish, fish-based spread), liver, roe, and shellfish. Total meat includes red meat (beef, pork, mutton, processed red meat), game and poultry. Other meat includes game and poultry. The total seafood, lean fish and fatty fish results have been published previously^8^.

In the evaluation of quintile categories of energy-adjusted seafood and meat intake, a higher risk of T2D was seen for intake of shellfish quintile five compared to quintile one (non-consumers) in the BMI group < 25 kg/m^2^, and a lower risk of T2D was observed for lean fish quintiles three to five compared to quintile one in the BMI group ≥ 25 kg/m^2^^8^ (Fig. 2). Similar findings were observed with use of energy-adjusted protein intake from seafood and meat (data not shown).

### The effect from intake of different meat and seafood sources on glucose tolerance and insulin secretion in mice

**Figure 3:**
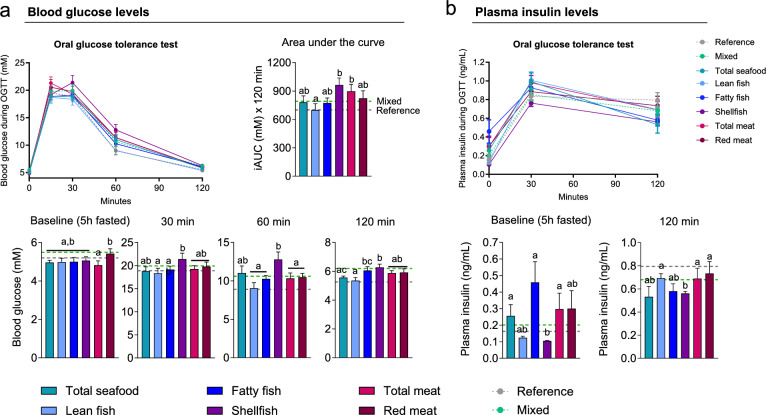
(**a**) Blood glucose levels during the oral glucose tolerance test (GTT), incremental area under the curve (iAUC) of the GTT and blood glucose levels for time point 0, 30, 60 and 120 min during OGTT performed in week 11 of feeding mice a low-fat reference diet and different experimental western diets based on protein from several sources (mixed), total seafood, lean fish, fatty fish, shellfish, total meat or red meat. (**b**) Plasma insulin levels (*n* = 9–10) during the GTT in week 11 and time points 0 and 120 min. The dotted lines represent the mean value of mice fed a low-fat reference diet and a western diet based on protein from several sources (mixed). Data are presented as mean ± standard error of the mean (SEM) and different letters denote significant differences (*P* < 0.05) by one-way ANOVA using uncorrected Fisher’s Least Significant Difference (LSD) multiple comparison, or nonparametric Kruskal–Wallis test using the uncorrected Dunn’s multiple comparison test for insulin levels at baseline (5 h fasted) and 120 min. All the data points in the figure are listed in Table S11 for each mouse.

To provide information on possible metabolic features governing the effect of the different protein sources in relation to the development of T2D, especially comparing lean fish with shellfish, 80 female C57BL/6J mice were fed isocaloric experimental diets where the protein sources comprised seafood, lean fish, fatty fish, shellfish, meat or red meat. In addition, a low-fat diet (reference) and a diet based on the average intake data from the participants in the MoBa study (mixed) were also included. After 11 weeks of feeding, the mice remained lean, with no difference in body mass, lean body mass or fat mass between the groups, despite an increased energy intake in mice fed red meat (Fig. S4, Table S10). Despite no differences in obesity development or body composition, the iAUC of glucose during the oral GTT and blood glucose levels measured 30, 60 and 120 min after glucose gavage were higher in shellfish-fed mice than in mice fed lean fish (Fig. 3, Table S11). The mice fed total meat also exhibited reduced glucose tolerance in comparison to the mice fed lean fish, whereas the other groups fed total seafood, fatty fish and red meat did not differ from mice fed lean fish. Plasma insulin levels measured in 6 h fasted mice and during the GTT after 30 min did not differ between mice fed shellfish and lean fish, but plasma insulin levels measured 120 min after the glucose load were reduced in mice fed shellfish compared to mice given lean fish and meat.

### Shellfish intake induces a distinct plasma metabolomic profile in mice

A comprehensive metabolomic analysis of plasma enabled the identification of 760 metabolites, of which 266 differed significantly in abundance between the dietary groups (q < 0.05) (Fig. S5, Tables S12, S13). Hierarchical clustering demonstrated a clear diet-specific metabolic signature in plasma elicited by intake of the different experimental western diets, comprising five distinct metabolic clusters in mice fed (I) fatty fish, (II) total seafood/lean fish, (III) shellfish, (IV) mixed and (V) meat (red meat and total meat).

**Figure 4:**
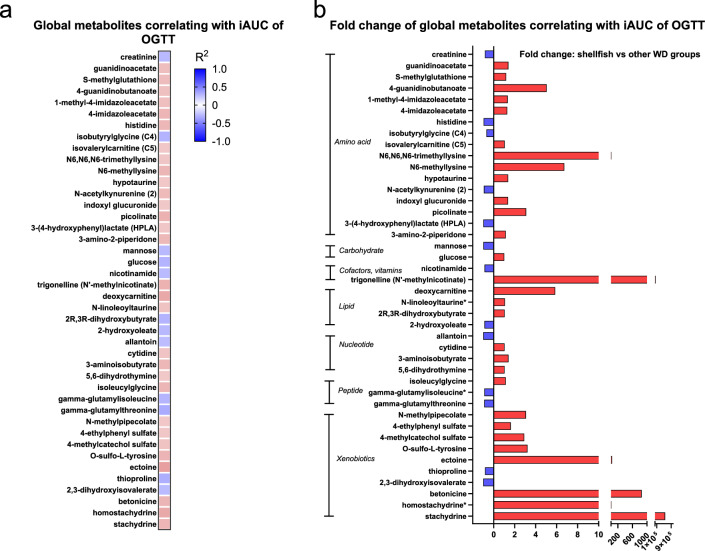
Illustrates the (**a**) correlation coefficient and (**b)** fold change of all the 42 known plasma metabolites significantly correlating with glucose tolerance (iAUC of the GTT curve) from the global metabolomics platform (rank regression, *P* < 0.05) in mice fed different experimental western diets (*n* = 8) based on protein from several sources (mixed), total seafood, lean fish, fatty fish, shellfish, total meat or red meat for 12 weeks. Color scale in (**a**) indicates the correlation coefficient for each metabolite. (**b**) The fold change between shellfish and the other western diet fed groups are demonstrated for all the positive (red) and negative (blue) correlated known metabolites, grouped into pathway. All correlating metabolites are listed in Table S14 including the pathway, correlation coefficient and *P* value from rank regression with iAUC. Statistical analyses were performed using the Qlucore omics explorer.

We identified 51 metabolites correlating with glucose tolerance (iAUC of the GTT, *P* < 0.05), of these 42 are known metabolites (Fig. 4). These metabolites included several different amino acids, carbohydrates, vitamins and cofactors, lipids, nucleotides, peptides and xenobiotics. All metabolites correlating with glucose tolerance demonstrated a diet dependent pattern (Fig. 4, Table S14, Fig. S6), and several of these metabolites were elevated in plasma from shellfish-fed mice.

### Plasma lipid species affected by the diet and associated with glucose tolerance in mice

The complex lipid platform identified 940 lipid species in plasma from the mice, with the abundances of 773 metabolites being significantly modulated by the different experimental western diets (Fig. S7, Tables S15, S16). Hierarchical clustering analysis of these 773 metabolites demonstrated distinct diet-specific clustering between mice fed diets based on fatty fish, lean fish or total seafood. Furthermore, 26 of the plasma lipid metabolites were significantly correlated with glucose tolerance (iAUC of the GTT) (Fig. S8, Table S17), including triacylglycerols containing saturated fatty acids, total phosphatidylethanolamine (PE), several PE species, a number of phosphatidylcholine (PC) species, monoacylglycerol (MAG) (22:6), lysophosphatidylcholine (LPC) (22:6) and cholesteryl esters (CE(22:6), CE(24:0)). Several of the lipid metabolites correlating with reduced glucose tolerance were also elevated in plasma from shellfish-fed mice compared to the other WD fed groups (Fig. S9, Table S17).

A distinct metabolomic profile characterized plasma from mice fed the shellfish-enriched diet (Fig. S10, Table S18). Most of these metabolites were upregulated in plasma from mice fed a shellfish-enriched diet compared to all the other dietary groups. The lipid specific platform identified 25 plasma metabolites exhibiting differences in abundance between the shellfish-enriched diet group and the other groups, including several phospholipids (two PCs and 20 PEs), two sphingomyelins and one hexosylceramide (Table S19).

## Discussion

In this large prospective cohort study of 60,777 women of childbearing age, we found that shellfish intake was associated with higher risk of T2D. In line with this, we demonstrated that lean female mice fed a shellfish-enriched diet exhibited reduced glucose tolerance and insulin secretion. In mice fed a shellfish-enriched diet, we observed specific changes in the abundance of several metabolites, which correlated with glucose tolerance. In the women, intake of lean fish was inversely associated with T2D, whereas no associations to T2D were observed for total seafood and fatty fish.

In our cohort, the risk for T2D was not associated with intake of meat in the adjusted analyses. This is in accordance with some cohort studies performed in Asia but not in US and Europe^1–4^. It has been speculated that the reason for this is that the Asian populations typically have lower meat intakes than the American and European populations. However, results from a recent meta-analysis of randomized controlled trials did not support a mechanistic link between red meat intake and T2D risk factors^5^. The women included in the current study had higher total meat (mean 152 g/d) and red meat (125 g/d) intakes than women in other European populations (total meat: mean 94 g/d, red meat: 40 g/d)^3^. In the present study, a higher risk of T2D was observed for intake of both total meat and red meat when BMI was not included as a covariate. This was also the case for the highest quintile of meat intake compared to the lowest quintile, indicating that these results were mediated by BMI. Similar effect modification by BMI has been observed in previous studies as well^3,28^.

Overweight and obesity play an important role in the onset of new cases of T2D, and obesity has been found to account for more than half of new T2D cases^29^. The current study demonstrates that shellfish intake was associated with increased risk of T2D in agreement with some^9–11^, but not all studies^12–15^. However, in the present study stratification demonstrated that the association between shellfish intake and increased risk of T2D was statistically significant in women with BMI < 25 kg/m^2^.

The preventive effect of high fish and seafood intake has been reported to be evident in populations with a high prevalence of obesity, and countries with a low fish and seafood intake have reported a higher prevalence of T2D^16^. A potential beneficial effect of fish intake in elderly in relation to prevention of impaired glucose tolerance has been reported^30^. Despite several studies demonstrating an association between fish and seafood intake and reduced T2D risk, the Nurses’ Health Study, including above 90,000 US women, did not find any association between fish intake and T2D risk^31^.

Longitudinal studies evaluating seafood intake and risk of T2D are inconsistent^7,11,14,15,32^, also within Europe where the average intake varies greatly^33,34^. The reasons may be related to several factors, including the high degree of variation in types and preparation of the ingested seafood^33,34^. The impact of metabolic intermediates in the progression to T2D has earlier been reported to have sex-specific effects on insulin action^35^ and women are found to be more susceptible to lipid-mediated β-cell lipotoxicity^36^, supporting a sex difference in diabetes development. Glucose-stimulated insulin secretion from the β-cells is also demonstrated to be dependent on availability of endogenous free fatty acids through hormone-sensitive lipase, and insulin secretion may also differ between genders^37^.

The cooking method such as frying has been demonstrated to impact T2D risk outcome^38^ and this has been suggested to influence the findings of increased T2D risk from shellfish intake in human cohorts^9,10,33^. Thus, the direct effect of shellfish intake per se is not necessarily detected from human cohort studies. The use of female mice as a model enabled us to detect the direct impact from shellfish intake per se, and the distinct plasma metabolite profile observed in mice fed the shellfish-based diet further supported the existence of a link between shellfish intake and reduced glucose tolerance. Finally, the animal experiment also supported an effect of shellfish intake on glucose-stimulated insulin secretion and a correlation with the distinct plasma metabolite profile.

**Figure 5:**
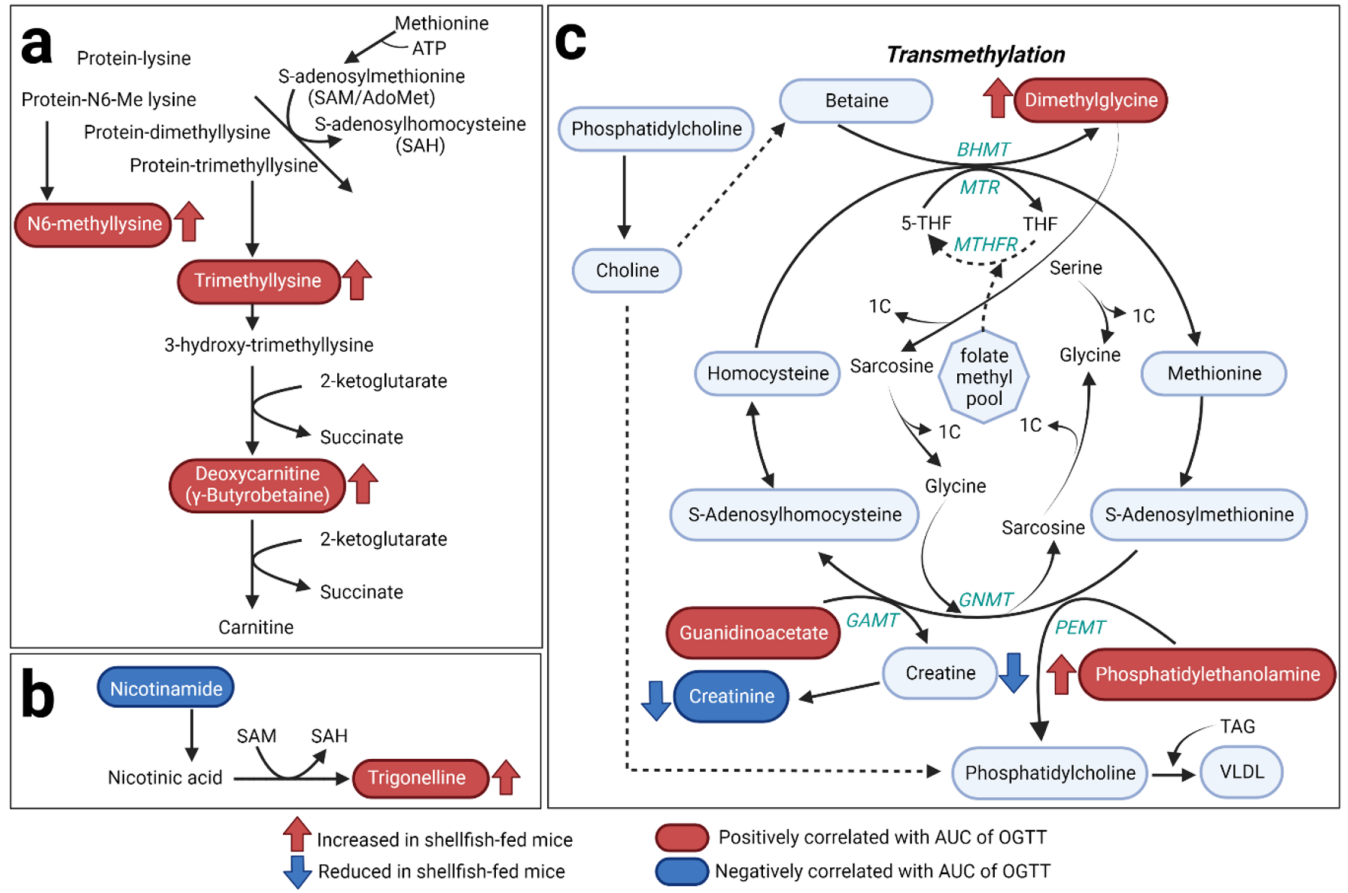
Illustration of affected metabolic pathways linked to methyl group metabolism, such as (**a**) carnitine metabolism, (**b**) nicotinamide metabolism and (**c**) production of s-adenosylmethionine through one-carbon metabolism fueled by folate, serine and glycine and associations with phosphatidylcholine and phosphatidylethanolamine metabolism. *THF* tetrahydrofolate, *MTR* 5-methyltetrahydrofolate-homocysteine methyltransferase, *BHMT* betaine-homocysteine S-methyltransferase, *MTHFR* methylenetetrahydrofolate reductase, *PEMT* phosphatidylethanolamine N-methyltransferase, *GNMT* Glycine-N-methyltransferase, *GAMT* guanidinoacetate N-methyltransferase. Created with BioRender.com.

Although studies have reported a positive association between shellfish intake and T2D risk^9,10,33^, the mechanisms still need to be elucidated. A recent study found that lean subjects with a plasma metabolome similar to obese subjects have increased risk for T2D^39^. The present study demonstrated that feeding mice shellfish induced a specific metabolic profile in plasma and led to a cluster of selectively regulated amino acids and lipid metabolites. Several metabolites exhibiting specific changes in abundance in response to shellfish intake and correlating with reduced glucose tolerance are involved in methyl group metabolism and metabolic pathways linked to methyl group metabolism, such as histidine metabolism, lysine metabolism, nicotinamide metabolism, PC and PE metabolism (Fig. 5, Tables S18, S19). Higher dietary intake of methyl donor nutrients has been found to reduce the likelihood of a person being metabolically unhealthy^40^, and supplementation with the methyl donor folate can improve glucose homeostasis and insulin resistance in humans^41^.

Mice fed the shellfish-enriched diet had increased circulating levels of guanidinoacetate and phosphatidylethanolamine, two major reported methyl consumers, due to the synthesis of creatine and PC^42^. S-adenosylmethionine (SAM) is a major methyl donor and involved in the important transmethylation pathways leading to the synthesis of creatine and PC^43^, which based on the metabolomic profile are affected by shellfish intake and glucose tolerance from our study (Fig. 5). The finding that the two major methyl consumers, guanidinoacetate and PE, both negatively correlated with glucose tolerance combined with the fact that especially the abundance of creatine was decreased in mice fed the shellfish-enriched diet indicated that SAM-dependent transmethylation may be affected, suggesting a potential mechanism of action. In addition, several PC species have been linked to reduced odds for abnormal fasting plasma glucose levels and HOMA-IR^44^. In our study, we detected five PC species (Fig. S9) positively correlated with glucose tolerance, all of them containing DHA (22:6n-3). Of note, PC 18:0/22:6 corresponds to PC C40:6, earlier associated with a decreased risk for abnormal HOMA-IR^44^ and T2D^45^.

In line with the proposed alterations in methyl group metabolism in shellfish-fed mice, we observed increased levels of trigonelline, a niacin catabolite methylated by the major methyldonor SAM. Carnitine metabolism was also demonstrated to be significantly affected upon shellfish intake, where several of the elevated plasma metabolites from shellfish-fed mice were carnitine precursors (Fig. 5). This is in agreement with a previous study reporting circulating levels of carnitine precursors as a risk factor for T2D in patients with coronary heart disease^46^.

Strengths of the current study are the inclusion of both human and animal data. The human data is based on a large population-based cohort comprising women with wide ranges of socioeconomic status and seafood and meat consumption, the availability of important pre-pregnancy risk factors and pregnancy-related complications, and the complete ascertainment of diabetic medication usage in a mandatory nationwide register during follow-up. The FFQ used has been validated and it was detailed enough to separately evaluate different types of seafood and meat. The finding that shellfish intake increases the risk of T2D in women was supported by a dietary intervention in female mice revealing plasma metabolites modulated by shellfish intake suggesting a potential mechanism of action. The carefully designed isocaloric mouse diets mimicked the average macronutrient composition calculated from the FFQs, and the protein sources were the sole varying factor.

This study has limitations. As the human study is based on observational data, we cannot rule out the possibility of unmeasured confounders, and causality cannot be inferred. Although FFQs are not suitable for precise dietary intake estimates, they are recognized for their suitability for rank-ordering study participants by dietary intakes for epidemiological investigations. Dietary intake data was only collected at one single point asked about habitual diet during the first half of pregnancy. Changes in dietary habits due to pregnancy cannot be excluded, but studies have shown that overall dietary habits remain relatively stable^47^. In MoBa, the majority of the participants (~ 80%) reported that fish intake was unchanged from before to during pregnancy^19^. Measurement error and misclassification would likely bias the results toward the null. In MoBa, dietary habits assessed during pregnancy have served as a proxy for long-term intake in several studies of diet-disease associations^8,48–50^. Furthermore, the mouse study demonstrates that even short time intake of a shellfish-based diet impacts glucose tolerance and is associated with distinct changes in plasma metabolite profiles. All mice in the animal trial were lean and does therefore not correspond in the totality to the women in MoBa. The low response rate (41%) in MoBa raises concerns regarding generalizability. However, an evaluation of potential selection bias in the MoBa study showed that the relative risks of eight exposure-outcome were similar to those obtained from the MBRN^51^. Another limitation includes the lack of diagnostic laboratory measurements and thereby a possibility of misclassification in the outcome of T2D. Furthermore, we could not identify non-pharmacologically treated T2D. Finally, it is unlikely that type 1 diabetes was included in the outcome given our exclusion criteria.

In conclusion, in this large prospective cohort study including women of childbearing age, we observed a positive association between intake of shellfish and risk of T2D in women, particularly women with BMI < 25 kg/m^2^. Intake of lean fish was inversely related to T2D, whereas no associations were found for intake of total seafood, fatty fish and different types of meat in the adjusted models. The experimental feeding-studies in mice revealed potential mechanisms comprising alterations in methyl group metabolism by which shellfish might impact glucose metabolism. This study contributes with new knowledge suggesting an increased T2D risk from intake of shellfish in women and providing a list of several distinct plasma metabolites found to be affected by shellfish intake and correlating with reduced glucose tolerance in mice. We suggest that the findings should be tested in other cohorts evaluating shellfish intake including diverse study groups and populations and further translational studies may add to the understanding of potential mechanisms.

### Supplementary Information

Supplementary Information.

Supplementary Tables.

## Data Availability

The data that support the findings of this study are available from The Norwegian Institute of Public Health and application to helsedata.no, but restrictions apply to the availability of these data which were used under license for the current study and therefore are not publicly available. Data are however available from the authors upon reasonable request and with permission of The Regional Committee for Medical and Health Research Ethics in Norway and an agreement with MoBa. All data from the animal experiment generated or analysed during this study are included in the published article and its supplementary files.

## Abbreviations

MoBa: The Norwegian Mother, Father, and Child Cohort Study
MBRN: Medical Birth Registry of Norway
NorPD: Norwegian Prescription Database
iAUC: Incremental area under the curve
CLP: Complex lipid panel
T2D: Type 2 diabetes
GTT: Glucose tolerance test
PCA: Principal component analysis
