# Supplementary material for: Intake of different types of seafood and meat and risk of type 2 diabetes in women: a prospective study supported by a dietary intervention in mice
Source: Sci Rep. 2024 Apr 18;14:8950. doi: 10.1038/s41598-024-59491-9 (PMC11026463; doi:10.1038/s41598-024-59491-9)
Supplement: Supplementary file 1 — Supplementary Information. [file 41598_2024_59491_MOESM1_ESM.docx]

**Intake of Different Types of Seafood and Meat and Risk of Type 2 Diabetes in Women: a Prospective Study Supported by a Dietary Intervention in Mice**

**Supplementary Material**

**Supplementary Tables**

**Table S1.** Composition of the experimental mouse diets.

| Component (g/kg diet) | Mixed diet | Total seafood | Lean fish | Fatty fish | Shellfish | Total meat | Red meat |
| --- | --- | --- | --- | --- | --- | --- | --- |
|  |  |  |  |  |  |  |  |
| **Added protein source** |  |  |  |  |  |  |  |
| Mixed protein mix* | 338.1 |  |  |  |  |  |  |
| Lean seafood protein mix^†^ |  | 117.5 | 199.1 |  |  |  |  |
| Fatty fish protein mix^‡^ |  | 23.7 |  | 308.9 |  |  |  |
| Shellfish protein mix^§^ |  | 92.7 |  |  | 215.6 |  |  |
| Total meat protein mix^\|\|^ |  |  |  |  |  | 277.4 |  |
| Red meat protein mix^¶^ |  |  |  |  |  |  | 302.3 |
|  |  |  |  |  |  |  |  |
| **Protein and amino acids** |  |  |  |  |  |  |  |
| From protein source^#^ | 160 | 160 | 160 | 160 | 160 | 160 | 160 |
| L-Cystine | 3.0 | 3.0 | 3.0 | 3.0 | 3.0 | 3.0 | 3.0 |
|  |  |  |  |  |  |  |  |
| **Fat** |  |  |  |  |  |  |  |
| From protein source^‡^ | 46.9 | 37.3 | 3.4 | 114.3 | 9.3 | 63.8 | 93.7 |
| Vegetable shortening | 33.9 | 36.9 | 47.0 | 13.7 | 45.2 | 28.9 | 19.9 |
| Milk fat | 33.9 | 36.9 | 47.0 | 13.7 | 45.2 | 28.9 | 19.9 |
| Lard | 33.9 | 36.9 | 47.0 | 13.7 | 45.2 | 28.9 | 19.9 |
| Soybean oil | 7.9 | 8.6 | 11.0 | 3.2 | 10.6 | 6.7 | 4.6 |
| Corn oil | 3.4 | 3.7 | 4.7 | 1.4 | 4.5 | 2.9 | 2.0 |
|  |  |  |  |  |  |  |  |
| **Other** |  |  |  |  |  |  |  |
| From protein source^‡^ | 131.2 | 36.6 | 35.7 | 34.6 | 46.3 | 53.6 | 48.6 |
| Sucrose | 295 | 295 | 295 | 295 | 295 | 295 | 295 |
| Corn starch | 153.3 | 247.9 | 248.8 | 249.9 | 238.1 | 230.9 | 235.9 |
| Cellulose | 50.0 | 50.0 | 50.0 | 50.0 | 50.0 | 50.0 | 50.0 |
| t-Butylhydroquinone | 0.01 | 0.01 | 0.01 | 0.01 | 0.01 | 0.01 | 0.01 |
| Mineral mix ^** 1^ | 35.0 | 35.0 | 35.0 | 35.0 | 35.0 | 35.0 | 35.0 |
| Vitamin mix ^†† 2^ | 10.0 | 10.0 | 10.0 | 10.0 | 10.0 | 10.0 | 10.0 |
| Choline Bitartrate | 2.5 | 2.5 | 2.5 | 2.5 | 2.5 | 2.5 | 2.5 |
|  |  |  |  |  |  |  |  |
| Analysed energy (kcal/100g) | 480 | 485 | 480 | 485 | 480 | 485 | 480 |

^*^27% gluten. 26% meat mix. 25.3% milk powder. 5.0% lean seafood mix. 3.7% peanut flour. 3.7% ground almonds. 3.7% soy. 2.5% fatty fish mix. 2.2% egg and 1.0% shellfish mix. ^†^50.0% cod. 20.0% pollack. 20.0% saithe. 10.0% lean tuna. ^‡^50.0% salmon. 40.0% mackerel. 10.0% herring. ^§^50.0% scampi. 20.0% crab. 20.0% scallops. 10.0% lobster. ^||^69.1% red meat mix. 28.8% chicken. 2.1% reindeer. ^¶^24.5 % sausage. 24.5% minced meat. 23.7% pork. 14.9% lamb. 13.3% steak. ^#^Protein sources were analysed for total fat and nitrogen allowing calculation of the contribution to total fat and protein content. ^** 1^AIN 93G MIN MIX (Special Diets Services, Essex, UK). ^†† 2^AIN 93-VX NCR95 COMPLIANT (Special Diets Services, Essex, UK).

**Table S2.** Protein sources used in the experimental diets.

| Protein source  (% of total protein content) | Mixed diet | Total seafood | Lean fish | Fatty fish | Shellfish | Total meat | Red meat |
| --- | --- | --- | --- | --- | --- | --- | --- |
|  |  |  |  |  |  |  |  |
| **Fatty fish** | **2.5** | **29.2** |  | **100** |  |  |  |
| Salmon | 1.3 | 14.6 |  | 50.0 |  |  |  |
| Mackerel | 0.6 | 7.3 |  | 40.0 |  |  |  |
| Herring | 0.6 | 7.3 |  | 10.0 |  |  |  |
|  |  |  |  |  |  |  |  |
| **Lean fish** | **5.0** | **60.0** | **100** |  |  |  |  |
| Cod | 2.5 | 30.0 | 50 |  |  |  |  |
| Saithe | 1.0 | 12.0 | 20 |  |  |  |  |
| Pollack | 1.0 | 12.0 | 20 |  |  |  |  |
| Lean tuna | 0.5 | 6.0 | 10 |  |  |  |  |
|  |  |  |  |  |  |  |  |
| **Shellfish** | **1.0** | **10.8** |  |  | **100** |  |  |
| Scampi | 0.5 | 3.0 |  |  | 50.0 |  |  |
| Scallops | 0.2 | 1.2 |  |  | 20.0 |  |  |
| Crab | 0.2 | 1.2 |  |  | 20.0 |  |  |
| Lobster | 0.1 | 0.6 |  |  | 10.0 |  |  |
|  |  |  |  |  |  |  |  |
| **Meat** | **26.0** |  |  |  |  | **100** | **100** |
| Beef | 2.4 |  |  |  |  | 9.2 | 13.3 |
| Pork | 4.3 |  |  |  |  | 16.4 | 23.7 |
| Mutton | 2.7 |  |  |  |  | 10.3 | 14.9 |
| Minced meat | 4.3 |  |  |  |  | 16.9 | 24.5 |
| Sausage | 4.3 |  |  |  |  | 2.1 | 24.5 |
| Reindeer | 0.5 |  |  |  |  | 28.9 |  |
| Chicken | 7.5 |  |  |  |  |  |  |
|  |  |  |  |  |  |  |  |
| **Egg/dairy** | **27.5** |  |  |  |  |  |  |
| Milk powder | 25.3 |  |  |  |  |  |  |
| Egg | 2.2 |  |  |  |  |  |  |
|  |  |  |  |  |  |  |  |
| **Vegetarian** | **38.1** |  |  |  |  |  |  |
| Gluten | 27 |  |  |  |  |  |  |
| Peanuts | 3.7 |  |  |  |  |  |  |
| Almonds | 3.7 |  |  |  |  |  |  |
| Soy | 3.7 |  |  |  |  |  |  |

**Table S3.** Amino acid composition of the experimental mouse diets.

| Amino acids (g/kg diet) | Mixed diet | Total seafood | Lean fish | Fatty fish | Shellfish | Total meat | Red meat |
| --- | --- | --- | --- | --- | --- | --- | --- |
|  |  |  |  |  |  |  |  |
| **Indispensable** |  |  |  |  |  |  |  |
| Histidine | 4.4 | 4.0 | 3.8 | 5.4 | 3.7 | 5.3 | 5.1 |
| Hydroxy-Proline | 0.7 | <0.6 | <0.6 | <0.6 | 0.6 | 1.4 | 1.9 |
| Isoleucine | 7.8 | 7.6 | 7.6 | 7.6 | 7.9 | 7.9 | 7.2 |
| Leucine | 14.4 | 13.5 | 13.8 | 13.4 | 14.2 | 14.0 | 13.2 |
| Lysine | 12.2 | 16.5 | 16.3 | 15.2 | 16.0 | 16.3 | 15.2 |
| Methionine | 5.3 | 6.1 | 6.5 | 6.8 | 6.7 | 6.1 | 5.5 |
| Phenylalanine | 8.3 | 6.9 | 7.1 | 7.5 | 7.7 | 7.1 | 6.7 |
| Threonine | 6.8 | 7.5 | 7.7 | 7.8 | 7.4 | 7.8 | 7.3 |
| Tryptophan | 1.5 | 1.8 | 1.7 | 1.8 | 1.7 | 1.7 | 1.6 |
| Valine | 8.6 | 8.2 | 8.2 | 8.6 | 7.6 | 8.2 | 7.8 |
|  |  |  |  |  |  |  |  |
| **Dispensable** |  |  |  |  |  |  |  |
| Alanine | 7.8 | 9.7 | 9.7 | 9.6 | 9.8 | 10.4 | 10.2 |
| Arginine | 9.0 | 9.7 | 10.0 | 9.5 | 12.1 | 10.5 | 10.0 |
| Aspartic acid | 14.8 | 18.1 | 18.1 | 15.8 | 19.4 | 17.2 | 16.2 |
| Glycine | 7.0 | 7.2 | 7.5 | 8.1 | 8.7 | 8.9 | 9.4 |
| Serine | 8.2 | 7.0 | 7.3 | 6.7 | 7.2 | 6.8 | 6.5 |
| Glutamic acid | 42.5 | 26.1 | 26.2 | 22.1 | 28.8 | 27.1 | 26.1 |
| Proline | 13.7 | 5.7 | 5.8 | 6.0 | 6.5 | 7.4 | 7.7 |
| Tyrosine | 6.0 | 5.2 | 5.5 | 5.6 | 5.5 | 5.2 | 4.9 |
| Cystein | 5.7 | 5.4 | 5.2 | 5.3 | 5.3 | 5.5 | 5.0 |
|  |  |  |  |  |  |  |  |
| Taurine | <0.6 | 0.9 | 1.0 | <0.6 | 1.7 | <0.6 | <0.6 |

**Table S4.** Fatty acid composition of the experimental diets.

| Fatty acids (g/kg diet) | Mixed diet | Total seafood | Lean fish | Fatty fish | Shellfish | Total meat | Red meat |
| --- | --- | --- | --- | --- | --- | --- | --- |
|  |  |  |  |  |  |  |  |
| **Sum saturated** | **51.9** | **50.6** | **56.0** | **34.7** | **61.2** | **44.6** | **60.2** |
| 08:0 | 0.3 | 0.3 | 0.4 | <0.01 | 0.5 | 0.3 | 0.2 |
| 10:0 | 0.9 | 0.9 | 1.2 | 0.3 | 1.4 | 0.9 | 0.6 |
| 12:0 | 1.2 | 1.2 | 1.5 | 0.5 | 1.7 | 1.2 | 0.8 |
| 14:0 | 4.5 | 5.7 | 5.3 | 6.2 | 5.8 | 5.3 | 4.1 |
| 15:0 | 0.4 | 0.5 | 0.5 | 0.5 | 0.6 | 0.6 | 0.5 |
| 16:0 | 27.8 | 26.6 | 29.0 | 19.3 | 31.5 | 36.0 | 32.2 |
| 17:0 | 0.5 | 0.6 | 0.5 | 0.3 | 0.6 | 0.9 | 0.9 |
| 18:0 | 15.3 | 13.8 | 16.6 | 6.8 | 18.1 | 22.4 | 20.3 |
| 20:0 | 0.5 | 0.6 | 0.6 | 0.5 | 0.6 | 0.5 | 0.4 |
| 22:0 | 0.3 | 0.3 | 0.3 | 0.2 | 0.3 | 0.2 | 0.2 |
| 24:0 | <0.01 | <0.01 | <0.01 | <0.01 | <0.01 | <0.01 | <0.01 |
|  |  |  |  |  |  |  |  |
| **Sum monousaturated**  14:1n-9 | **56.0**  0.3 | **52.1**  0.3 | **43.7**  0.3 | **65.1**  0.2 | **47.8**  0.3 | **62.0**  0.6 | **55.2**  0.3 |
| 16:1n-9 | 0.4 | 0.4 | 0.4 | 0.4 | 0.4 | 2.7 | 0.6 |
| 16:1n-7 | 1.6 | 2.2 | 1.5 | 3.5 | 1.9 | 2.4 | 2.8 |
| 18:1n-11 | <0.01 | <0.01 | <0.01 | <0.01 | <0.01 | <0.01 | <0.01 |
| 18:1n-9 | 49.9 | 38.8 | 38.0 | 37.0 | 41.3 | 51.8 | 47.9 |
| 18:1n-7  20:1n-11 | 2.9  <0.01 | 3.2  0.3 | 2.8  <0.01 | 3.5  0.6 | 3.3  <0.01 | 3.2  0.2 | 3.1  <0.01 |
| 20:1n-9 | 0.6 | 3.0 | 0.5 | 8.2 | 0.4 | 0.5 | 0.5 |
| 20:1n-7 | <0.01 | <0.01 | <0.01 | 0.2 | <0.01 | <0.01 | <0.01 |
| 22:1n-11 | 0.4 | 3.3 | 0.2 | 9.8 | 0.2 | <0.01 | <0.01 |
| 22:1n-9 | <0.01 | 0.4 | <0.01 | 0.9 | <0.01 | 0.3 | <0.01 |
| 24:1n-9 | <0.01 | 0.3 | <0.01 | 0.7 | <0.01 | 0.3 | <0.01 |
|  |  |  |  |  |  |  |  |
| **Sum polyunsaturated** | **22.5** | **25.3** | **17.4** | **39.8** | **19.6** | **35.0** | **12.5** |
|  |  |  |  |  |  |  |  |
| **Sum n-6** | **20.1** | **13.9** | **13.5** | **13.7** | **15.5** | **14.9** | **10.8** |
| 18:2n-6 | 19.8 | 13.0 | 13.1 | 11.5 | 14.8 | 14.2 | 10.3 |
| 18:3n-6  20:2n-6 | <0.01  0.2 | <0.01  0.4 | <0.01  0.2 | <0.01  0.8 | <0.01  0.3 | <0.01  0.2 | <0.01  0.2 |
| 20:3n-6 | <0.01 | <0.01 | <0.01 | 0.2 | <0.01 | <0.01 | <0.01 |
| 20:4n-6 (ARA*) | 0.3 | 0.5 | 0.2 | 0.9 | 0.5 | 0.4 | 0.4 |
| 22:4n-6 | <0.01 | <0.01 | <0.01 | <0.01 | <0.01 | <0.01 | <0.01 |
| 22:5n-6 | <0.01 | <0.01 | <0.01 | 0.3 | <0.01 | <0.01 | <0.01 |
|  |  |  |  |  |  |  |  |
| **Sum n-3** | **2.0** | **11.1** | **3.6** | **25.9** | **3.8** | **1.2** | **1.1** |
| 16:4n-3 | <0.01 | <0.01 | <0.01 | 0.2 | <0.01 | <0.01 | <0.01 |
| 18:3n-3 | 1.4 | 2.3 | 1.1 | 4.8 | 1.2 | 1.3 | 1.1 |
| 18:4n-3 | <0.01 | 0.9 | <0.01 | 2.7 | <0.01 | <0.01 | <0.01 |
| 20:4n-3 | <0.01 | 0.4 | <0.01 | 1.0 | <0.01 | <0.01 | <0.01 |
| 20:5n-3 (EPA**^†^**) | 0.2 | 2.5 | 0.7 | 5.7 | 1.4 | <0.01 | <0.01 |
| 21:5n-3 | <0.01 | <0.01 | <0.01 | 0.4 | <0.01 | <0.01 | <0.01 |
| 22:5n-3 (DPA^‡^) | <0.01 | 0.6 | <0.01 | 1.5 | <0.01 | <0.01 | <0.01 |
| 22:6n-3 (DHA ^§^) | 0.4 | 4.5 | 1.8 | 9.3 | 1.1 | <0.01 | <0.01 |
| 24:5n-3 | <0.01 | <0.01 | <0.01 | 0.3 | <0.01 | <0.01 | <0.01 |
|  |  |  |  |  |  |  |  |
| **Sum unidentified** | **16.2** | **16.1** | **20.6** | **8.4** | **22.0** | **16.2** | **12.4** |
| **Sum identified** | **131** | **128** | **118** | **140** | **129** | **144** | **129** |

*ARA; Arachidonic acid. **^†^**EPA; Eicosapentaenoic acid. ^‡^DPA; Docosapentaenoic acid. ^§^DHA; Docosahexaenoic acid.

**Table S5.** Characteristics and the energy-adjusted daily dietary intake by quintiles of shellfish intake in 60,777 women with 681 T2D events.

|  | Quintiles of shellfish intake (g/1000 kcal) | | | | |
| --- | --- | --- | --- | --- | --- |
|  | Q1 (0.0) | Q2 (>0.0-1.1) | Q3 (>1.0-1.8) | Q4 (>1.8-3.0) | Q5 (>3.0) |
| *N* | 21,170 | 9,901 | 9,901 | 9,904 | 9,901 |
|  | Mean (SD) | Mean (SD) | Mean (SD) | Mean (SD) | Mean (SD) |
|  | Median (P5, P95) | Median (P5, P95) | Median (P5, P95) | Median (P5, P95) | Median (P5, P95) |
| Follow-up, years | 7.2 (1.5) | 7.1 (1.5) | 7.1 (1.5) | 7.1 (1.5) | 7.1 (1.6) |
|  | 7.5 (4.5, 9.5) | 7.5 (4.5, 9.5) | 7.5 (4.5, 9.5) | 7.5 (4.5, 9.5) | 7.5 (4.5, 9.5) |
| Age at delivery, years | 29.9 (4.7) | 30.8 (4.4) | 30.9 (4.2) | 30.8 (4.4) | 30.7 (4.5) |
|  | 30.0 (22.0, 38.0) | 31.0 (23.0, 38.0) | 31.0 (24.0, 38.0) | 31.0 (24.0, 38.0) | 31.0 (23.0, 38.0) |
| Height, cm | 1.68 (0.06) | 1.68 (0.05) | 1.68 (0.05) | 1.68 (0.05) | 1.68 (0.06) |
|  | 1.68 (1.58, 1.78) | 1.68 (1.59, 1.78) | 1.68 (1.59, 1.78) | 1.68 (1.59, 1.78) | 1.68 (1.58, 1.79) |
| *Pre-pregnancy variables* |  |  |  |  |  |
| Weight, kg | 68.4 (13.3) | 67.8 (12.5) | 67.8 (12.3) | 67.9 (12.3) | 68.0 (12.9) |
|  | 66.0 (52.0, 94.0) | 65.0 (52.0, 92.0) | 65.0 (52.0, 92.0) | 65.0 (52.0, 91.0) | 65.0 (52.0, 93.0) |
| BMI, kg/m^2^ | 24.2 (4.4) | 23.9 (4.2) | 23.9 (4.0) | 23.9 (4.0) | 24.1 (4.2) |
|  | 23.2 (18.9, 33.1) | 23.0 (18.9, 32.1) | 23.0 (18.9, 31.8) | 23.0 (19.0, 32.1) | 23.1 (19.0, 32.7) |
|  | % | % | % | % | % |
| BMI <25 kg/m^2^ (%) | 66.3 | 69.7 | 69.6 | 69.4 | 68.5 |
| Married/partner (%) | 96.3 | 97.2 | 97.2 | 97.6 | 96.9 |
| Daily smoking (%) | 18.5 | 15.3 | 13.6 | 13.2 | 15.9 |
| Physical activity ≥3 times/wk (%) | 44.6 | 48.7 | 50.3 | 50.3 | 49.7 |
| Education ≥17 years (%) | 23.1 | 29.2 | 32.1 | 31.6 | 30.6 |
| *Pregnancy variables* |  |  |  |  |  |
| Gestational diabetes (%) | 0.9 | 1.0 | 0.9 | 1.0 | 1.1 |
| Gestational hypertension (%) | 2.2 | 1.9 | 2.1 | 2.1 | 2.1 |
| Preeclampsia (%) | 3.8 | 3.8 | 3.5 | 3.5 | 3.8 |
|  | Mean (SD) | Mean (SD) | Mean (SD) | Mean (SD) | Mean (SD) |
| *Daily intake* | Median (P5, P95) | Median (P5, P95) | Median (P5, P95) | Median (P5, P95) | Median (P5, P95) |
| Energy, kcal | 2261 (600) | 2487 (616) | 2369 (558) | 2262 (546) | 2110 (559) |
|  | 2184 (1409, 3413) | 2419 (1592, 3635) | 2303 (1569, 3430) | 2194 (1478, 3278) | 2034 (1331, 3180) |
| Total seafood^*^, g/1000 kcal | 14.4 (10.4) | 14.0 (8.7) | 15.8 (8.6) | 17.4 (8.7) | 22.3 (10.5) |
|  | 12.8 (0.00, 33.5) | 12.5 (2.8, 29.9) | 14.3 (5.1, 31.8) | 15.9 (6.3, 33.5) | 20.4 (9.3, 42.0) |
| Lean fish, g/1000 kcal | 9.3 (7.5) | 8.0 (5.7) | 8.6 (5.5) | 9.1 (5.7) | 10.4 (6.9) |
|  | 8.1 (0.00, 22.9) | 7.0 (0.65, 18.5) | 7.7 (1.6, 18.9) | 8.2 (1.8, 19.9) | 9.1 (1.7, 23.2) |
| Fatty fish, g/1000 kcal | 4.4 (5.9) | 4.6 (5.2) | 5.0 (5.3) | 5.1 (5.2) | 5.8 (5.6) |
|  | 2.6 (0.00, 15.7) | 3.0 (0.00, 14.4) | 3.4 (0.00, 15.1) | 3.7 (0.00, 14.8) | 4.4 (0.00, 16.3) |
| Shellfish, g/1000 kcal | 0.00 (0.00) | 0.65 (0.25) | 1.4 (0.21) | 2.3 (0.34) | 5.3 (3.2) |
|  | 0.00 (0.00, 0.00) | 0.66 (0.23, 1.0) | 1.4 (1.0, 1.7) | 2.3 (1.8, 2.9) | 4.3 (3.1, 10.9) |
| Total meat^†^, g/1000 kcal | 73.0 (25.9) | 67.3 (22.1) | 68.1 (21.6) | 69.6 (22.7) | 70.3 (23.9) |
|  | 70.9 (35.1, 119) | 65.1 (36.3, 106) | 66.2 (37.0, 106) | 67.4 (36.4, 110) | 68.6 (34.5, 112) |
| Red meat, g/1000 kcal | 60.8 (24.4) | 55.4 (20.5) | 55.5 (20.3) | 56.3 (21.2) | 56.4 (22.3) |
|  | 58.5 (24.9, 104) | 53.2 (26.0, 92.2) | 53.9 (26.1, 91.6) | 54.2 (25.7, 94.7) | 54.2 (23.8, 96.0) |
| Other meat^‡^, g/1000 kcal | 12.2 (9.5) | 11.6 (8.0) | 12.5 (8.0) | 13.2 (8.4) | 13.9 (9.2) |
|  | 10.0 (0.00, 30.1) | 10.1 (2.5, 27.6) | 10.8 (2.6, 28.0) | 11.5 (2.7, 29.3) | 12.0 (2.0, 31.1) |
| Milk/dairy, g/1000 kcal | 219 (133) | 230 (133) | 221 (131) | 221 (131) | 212 (132) |
|  | 201 (38.5, 482) | 214 (50.0, 480) | 206 (46.8, 463) | 203 (48.2, 460) | 194 (42.3, 458) |
| Bread/cereals/pasta, g/1000 kcal | 134 (37.9) | 134 (35.1) | 135 (34.4) | 134 (35.2) | 132 (37.3) |
|  | 133 (73.5, 197) | 132 (78.4, 193) | 134 (81.2, 194) | 133 (78.3, 193) | 131 (70.8, 194) |
| Eggs, g/1000 kcal | 9.9 (7.4) | 11.0 (7.2) | 11.2 (7.0) | 11.8 (7.5) | 12.6 (8.0) |
|  | 7.7 (2.3, 24.1) | 9.0 (3.3, 24.7) | 9.3 (3.5, 24.7) | 9.8 (3.6, 26.1) | 10.6 (3.7, 27.7) |
| Vegetables/fruits/nuts, g/1000 kcal | 350 (167) | 355 (151) | 363 (152) | 366 (151) | 372 (163) |
|  | 323 (131, 655) | 336 (147, 627) | 341 (156, 632) | 346 (159, 635) | 348 (155, 672) |
| Cholesterol, mg/1000 kcal | 103 (28.1) | 103 (25.8) | 107 (25.4) | 111 (27.4) | 120 (31.2) |
|  | 99.5 (68.2, 151) | 99.7 (70.9, 148) | 102 (75.3, 151) | 106 (78.1, 160) | 114 (83.0, 176) |
| Fibre, g/1000 kcal | 13.4 (3.2) | 13.4 (3.0) | 13.7 (2.9) | 13.7 (3.0) | 13.7 (3.2) |
|  | 13.1 (8.6, 19.1) | 13.2 (9.0, 18.6) | 13.4 (9.4, 18.9) | 13.4 (9.3, 19.0) | 13.4 (9.0, 19.4) |
| Added sugar, E% | 10.3 (5.2) | 10.5 (4.8) | 10.2 (4.3) | 10.0 (4.2) | 9.9 (4.4) |
|  | 9.4 (3.8, 19.7) | 9.8 (4.5, 19.2) | 9.5 (4.5, 18.0) | 9.4 (4.4, 17.5) | 9.2 (4.1, 18.0) |
| Protein, E% | 15.3 (2.1) | 15.2 (2.0) | 15.4 (1.9) | 15.7 (1.9) | 16.0 (2.0) |
|  | 15.3 (11.9, 19.0) | 15.1 (12.0, 18.7) | 15.4 (12.4, 18.8) | 15.6 (12.6, 19.0) | 15.9 (12.8, 19.6) |
| Carbohydrate, E% | 53.8 (4.9) | 53.6 (4.5) | 53.5 (4.4) | 53.2 (4.4) | 52.9 (4.6) |
|  | 53.7 (46.3) | 53.6 (46.3, 61.2) | 53.5 (46.4, 60.6) | 53.2 (46.0, 60.5) | 52.9 (45.4, 60.4) |
| Total fat, E% | 30.5 (4.6) | 30.8 (4.4) | 30.7 (4.3) | 30.8 (4.3) | 30.7 (4.4) |
|  | 30.4 (23.1, 38.0) | 30.7 (23.7, 38.2) | 30.6 (23.8, 37.9) | 30.6 (23.8, 38.1) | 30.7 (23.5, 38.0) |
| Saturated fat, E% | 11.8 (2.1) | 11.9 (2.0) | 11.8 (1.9) | 11.7 (2.0) | 11.6 (2.0) |
|  | 11.7 (8.5, 15.5) | 11.7 (8.7, 15.4) | 11.7 (8.7, 15.2) | 11.6 (8.7, 15.1) | 11.6 (8.5, 15.0) |
| Monounsaturated fat, E% | 9.7 (1.8) | 9.9 (1.8) | 9.9 (1.7) | 10.0 (1.8) | 10.0 (1.8) |
|  | 9.6 (6.9, 12.7) | 9.8 (7.2, 13.0) | 9.8 (7.3, 13.0) | 9.8 (7.3, 13.0) | 9.9 (7.2, 13.1) |
| Polyunsaturated fat, E% | 5.3 (3.6, 8.6) | 5.5 (3.7, 8.7) | 5.5 (3.8, 8.5) | 5.5 (3.8, 8.6) | 5.5 (3.8, 8.6) |
| *Type 2 diabetes* |  |  |  |  |  |
| Follow-up time, years | 7.2 (1.5) | 7.1 (1.5) | 7.1 (1.5) | 7.1 (1.5) | 7.1 (1.6) |
|  | 7.5 (4.5, 9.5) | 7.5 (4.5, 9.5) | 7.5 (4.5, 9.5) | 7.5 (4.5, 9.5) | 7.5 (4.5, 9.5) |
|  | % | % | % | % | % |
| Events | 1.2 | 1.0 | 1.0 | 0.9 | 1.4 |

Data are mean (SD), median (5^th^, 95^th^ percentile (P5, P95)) or percentage.

*Total seafood includes lean and fatty fish (salt and freshwater fish, fish-based spread), liver, roe, and shellfish.

**^†^**Total meat includes red meat (beef, pork, mutton, processed red meat), game, poultry.

^‡^Other meat includes game, poultry.

**Table S6.** The energy-adjusted daily intake of protein from different sources (n=60,777).

|  | Total cohort | |
| --- | --- | --- |
|  | Mean (SD) | Median (P5, P95) |
| Protein total seafood^*^, g/1000 kcal | 3.2 (2.0) | 2.9 (0.53, 6.7) |
| Protein lean fish, g/1000 kcal | 1.9 (1.4) | 1.6 (0.00, 4.3) |
| Protein fatty fish, g/1000 kcal | 0.92 (0.96) | 0.68 (0.00, 2.6) |
| Protein shellfish, g/1000 kcal | 0.34 (0.48) | 0.21 (0.00, 1.1) |
| Protein total meat^†^, g/1000 kcal | 10.0 (3.4) | 9.6 (5.1, 16.0) |
| Protein red meat, g/1000 kcal | 6.9 (2.6) | 6.6 (3.0, 11.4) |
| Protein other meat^‡^, g/1000 kcal | 3.1 (2.3) | 2.6 (0.31, 7.4) |
| Protein milk/dairy, g/1000 kcal | 9.1 (4.6) | 8.6 (2.6, 17.4) |
| Protein bread/cereals/pasta, g/1000 kcal | 9.8 (2.8) | 9.7 (5.0, 14.3) |
| Protein eggs, g/1000 kcal | 0.83 (0.74) | 0.59 (0.21, 2.1) |
| Protein vegetables/fruits/nuts, g/1000 kcal | 4.1 (1.6) | 3.8 (2.0, 7.0) |

Data are mean (SD) and median (5^th^, 95^th^ percentile (P5, P95)) or percentage. *Total seafood includes lean and fatty fish (salt and freshwater fish, fish-based spread), liver, roe, and shellfish. **^†^**Total meat includes red meat (beef, pork, mutton, processed red meat), game, and poultry. ^‡^Other meat includes game and poultry.

**Table S7.** HRs (95% CIs) for incident type 2 diabetes (T2D) by energy-adjusted protein intake from seafood and meat in 60,777 women with 681 T2D events.

|  | Model 1^*^ | Model 2^†^ | Model 3^‡^ |
| --- | --- | --- | --- |
|  |  |  |  |
| Protein total seafood^§^ (g/1000 kcal) | 0.97 (0.93-1.01); | 0.99 (0.95-1.02); | 0.98 (0.94-1.02); |
|  | *P* = 0.107 | *P* = 0.439 | *P* = 0.388 |
|  |  |  |  |
| Protein lean fish (g/1000 kcal) | 0.93 (0.87-0.98); | 0.94 (0.89-0.99); | 0.94 (0.89-0.99); |
|  | *P* = 0.013 | *P* = 0.043 | *P* = 0.027 |
|  |  |  |  |
| Protein fatty fish (g/1000 kcal) | 0.95 (0.87-1.03); | 0.99 (0.91-1.07); | 0.99 (0.92-1.08); |
|  | *P* = 0.198 | *P* = 0.796 | *P* = 0.873 |
|  |  |  |  |
| Protein shellfish (g/1000 kcal) | 1.18 (1.05-1.33); | 1.20 (1.06-1.35); | 1.20 (1.07-1.35); |
|  | *P* = 0.006 | *P* = 0.003 | *P* = 0.002 |
|  |  |  |  |
| Protein total meat^\|\|^ (g/1000 kcal) | 1.05 (1.03-1.08); | 1.01 (0.98-1.04); | 1.01 (0.98-1.04); |
|  | *P* <0.001 | *P* = 0.492 | *P* = 0.510 |
|  |  |  |  |
| Protein red meat (g/1000 kcal) | 1.08 (1.05-1.12); | 1.01 (0.98-1.04); | 1.01 (0.98-1.04); |
|  | *P* <0.001 | *P* = 0.559 | *P* = 0.663 |
|  |  |  |  |
| Protein other meat^¶^ (g/1000 kcal) | 0.99 (0.96-1.03); | 1.00 (0.97-1.04); | 1.01 (0.97-1.04); |
|  | *P* = 0.738 | *P* = 0.797 | *P* = 0.700 |

Data are HR (95% CI). ^*^Adjusted for energy intake and age. ^†^Adjusted for energy intake, age, pre-pregnancy BMI, gestational diabetes and gestational hypertension including preeclampsia. ^‡^Adjusted for energy intake, age, pre-pregnancy BMI, gestational diabetes, gestational hypertension including preeclampsia, maternal education, pre-pregnancy smoking and dietary fibre. ^§^Total seafood includes lean and fatty fish (salt and freshwater fish, fish-based spread), liver, roe and shellfish. ^||^Total meat includes red meat (beef, pork, mutton, processed red meat), game and poultry. ^¶^Other meat includes game and poultry.

**Table S8.** HRs (95% CIs) for incident type 2 diabetes (T2D) by energy-adjusted seafood and meat intake stratified by BMI <25 vs ≥25 kg/m^2^ in 60,777 women with 681 T2D events.

|  | Model 1^*^ | |  | Model 2^†^ | |  | Model 3^‡^ | |
| --- | --- | --- | --- | --- | --- | --- | --- | --- |
|  | BMI <25 kg/m^2^ | BMI ≥25 kg/m^2^ |  | BMI <25 kg/m^2^ | BMI ≥25 kg/m^2^ |  | BMI <25 kg/m^2^ | BMI ≥25 kg/m^2^ |
|  |  |  |  |  |  |  |  |  |
| *N* | 41,498 | 19,279 |  | 41,498 | 19,279 |  | 41,498 | 19,279 |
|  |  |  |  |  |  |  |  |  |
| *n* with T2D | 159 | 522 |  | 159 | 522 |  | 159 | 522 |
|  |  |  |  |  |  |  |  |  |
| Total seafood^§^ (25 g/1000 kcal) | 1.20 (0.82-1.77); | 0.84 (0.67-1.06); |  | 1.21 (0.82-1.80); | 0.83 (0.66-1.04); |  | 1.18 (0.80-1.76); | 0.83 (0.66-1.04); |
|  | *P* = 0.356 | *P* = 0.141 |  | *P* = 0.333 | *P* = 0.103 |  | *P* = 0.410 | *P* = 0.103 |
|  |  |  |  |  |  |  |  |  |
| Lean fish (25 g/1000 kcal) | 1.06 (0.57-1.96); | 0.64 (0.45-0.91); |  | 1.12 (0.60-2.07); | 0.62 (0.44-0.88); |  | 1.04 (0.56-1.93); | 0.61 (0.43-0.86); |
|  | *P* = 0.861 | *P* = 0.012 |  | *P* = 0.726 | *P* = 0.007 |  | *P* = 0.896 | *P* = 0.005 |
|  |  |  |  |  |  |  |  |  |
| Fatty fish (25 g/1000 kcal) | 0.87 (0.42-1.82); | 0.93 (0.62-1.38); |  | 0.89 (0.42-1.89); | 0.94 (0.63-1.38); |  | 0.90 (0.42-1.90); | 0.96 (0.65-1.42); |
|  | *P* = 0.716 | *P* = 0.703 |  | *P* = 0.769 | *P* = 0.737 |  | *P* = 0.776 | *P* = 0.847 |
|  |  |  |  |  |  |  |  |  |
| Shellfish (5 g/1000 kcal) | 1.35 (1.18-1.56); | 1.12 (0.95-1.32); |  | 1.32 (1.12-1.56); | 1.10 (0.93-1.30); |  | 1.31 (1.11-1.55); | 1.11 (0.94-1.30); |
|  | *P* <0.001 | *P* = 0.174 |  | *P* = 0.001 | *P* = 0.262 |  | *P* = 0.001 | *P* = 0.234 |
|  |  |  |  |  |  |  |  |  |
| Total meat^\|\|^ (25 g/1000 kcal) | 1.04 (0.84-1.28); | 1.07 (0.96-1.20); |  | 1.02 (0.83-1.25); | 1.03 (0.92-1.16); |  | 1.04 (0.84-1.27); | 1.03 (0.91-1.15); |
|  | *P* = 0.739 | *P* = 0.238 |  | *P* = 0.871 | *P* = 0.568 |  | *P* = 0.737 | *P* = 0.674 |
|  |  |  |  |  |  |  |  |  |
| Red meat (25 g/1000 kcal) | 1.08 (0.88-1.32); | 1.05 (0.94-1.17); |  | 1.06 (0.87-1.30); | 1.01 (0.90-1.12); |  | 1.08 (0.88-1.33); | 0.99 (0.89-1.11); |
|  | *P* = 0.471 | *P* = 0.392 |  | *P* = 0.550 | *P* = 0.925 |  | *P* = 0.458 | *P* = 0.872 |
|  |  |  |  |  |  |  |  |  |
| Other meat^¶^ (25 g/1000 kcal) | 0.79 (0.48-1.31); | 1.09 (0.85-1.40); |  | 0.77 (0.46-1.27); | 1.14 (0.89-1.47); |  | 0.79 (0.48-1.29); | 1.18 (0.92-1.51); |
|  | *P* = 0.357 | *P* = 0.509 |  | *P* = 0.299 | *P* = 0.287 |  | *P* = 0.341 | *P* = 0.187 |

Data are HR (95% CI) unless otherwise indicated. ^*^Adjusted for energy intake and age. ^†^Adjusted for energy intake, age, gestational diabetes and gestational hypertension including preeclampsia. ^‡^Adjusted for energy intake, age, gestational diabetes, gestational hypertension including preeclampsia, maternal education, pre-pregnancy smoking and dietary fibre. ^§^Total seafood includes lean and fatty fish (salt and freshwater fish, fish-based spread), liver, roe and shellfish. ^||^Total meat includes red meat (beef, pork, mutton, processed red meat), game and poultry. ^¶^Other meat includes game and poultry. The total seafood, lean fish and fatty fish results have been published previously^8^.

**Table S9.** HRs (95% CIs) for incident type 2 diabetes (T2D) by energy-adjusted protein intake from seafood and meat stratified by pre-pregnancy BMI <25 vs ≥25 kg/m^2^ in 60,777 women with 681 T2D events.

|  | Model 1^*^ | |  | Model 2^†^ | |  | Model 3^‡^ | |
| --- | --- | --- | --- | --- | --- | --- | --- | --- |
|  | BMI <25 kg/m^2^ | BMI ≥25 kg/m^2^ |  | BMI <25 kg/m^2^ | BMI ≥25 kg/m^2^ |  | BMI <25 kg/m^2^ | BMI ≥25 kg/m^2^ |
|  |  |  |  |  |  |  |  |  |
| *N* | 41,498 | 19,279 |  | 41,498 | 19,279 |  | 41,498 | 19,279 |
|  |  |  |  |  |  |  |  |  |
| *n* with type T2D | 159 | 522 |  | 159 | 522 |  | 159 | 522 |
|  |  |  |  |  |  |  |  |  |
| Protein total seafood^§^ (g/1000 kcal) | 1.05 (0.97-1.14); | 0.96 (0.92-1.01); |  | 1.05 (0.97-1.14); | 0.96 (0.92-1.01); |  | 1.05 (0.97-1.14); | 0.96 (0.92-1.01); |
|  | *P* = 0.201 | *P* = 0.117 |  | *P* = 0.199 | *P* = 0.082 |  | *P* = 0.259 | *P* = 0.085 |
|  |  |  |  |  |  |  |  |  |
| Protein lean fish (g/1000 kcal) | 1.04 (0.92-1.16); | 0.92 (0.86-0.98); |  | 1.04 (0.93-1.17); | 0.91 (0.85-0.97); |  | 1.03 (0.92-1.16); | 0.91 (0.85-0.97); |
|  | *P* = 0.569 | *P* = 0.012 |  | *P* = 0.467 | *P* = 0.006 |  | *P* = 0.629 | *P* = 0.004 |
|  |  |  |  |  |  |  |  |  |
| Protein fatty fish (g/1000 kcal) | 0.96 (0.81-1.14); | 0.98 (0.90-1.08); |  | 0.96 (0.81-1.15); | 0.99 (0.90-1.09); |  | 0.97 (0.81-1.15); | 1.00 (0.91-1.09); |
|  | *P* = 0.640 | *P* = 0.723 |  | *P* = 0.686 | *P* = 0.828 |  | *P* = 0.704 | *P* = 0.958 |
|  |  |  |  |  |  |  |  |  |
| Protein shellfish (g/1000 kcal) | 1.34 (1.18-1.51); | 1.12 (0.96-1.31); |  | 1.31 (1.13-1.52); | 1.10 (0.94-1.29); |  | 1.30 (1.12-1.51); | 1.10 (0.95-1.30); |
|  | *P* <0.001 | *P* = 0.155 |  | *P* <0.001 | *P* = 0.235 |  | *P* <0.001 | *P* = 0.211 |
|  |  |  |  |  |  |  |  |  |
| Protein total meat^\|\|^ (g/1000 kcal) | 1.00 (0.95-1.06); | 1.03 (1.00-1.07); |  | 1.00 (0.94-1.06); | 1.03 (0.99-1.06); |  | 1.00 (0.95-1.06); | 1.03 (0.99-1.06); |
|  | *P* = 0.920 | *P* = 0.053 |  | *P* = 0.958 | *P* = 0.106 |  | *P* = 0.904 | *P* = 0.113 |
|  |  |  |  |  |  |  |  |  |
| Protein red meat (g/1000 kcal) | 1.03 (0.96-1.11); | 1.03 (0.99-1.07); |  | 1.03 (0.96-1.10); | 1.02 (0.98-1.06); |  | 1.03 (0.97-1.11); | 1.01 (0.97-1.05); |
|  | *P* = 0.367 | *P* = 0.116 |  | *P* = 0.427 | *P* = 0.385 |  | *P* = 0.349 | *P* = 0.548 |
|  |  |  |  |  |  |  |  |  |
| Protein other meat^¶^ (g/1000 kcal) | 0.97 (0.89-1.05); | 1.02 (0.98-1.06); |  | 0.96 (0.90-1.04); | 1.02 (0.98-1.06); |  | 0.97 (0.89-1.05); | 1.03 (0.99-1.07); |
|  | *P* = 0.399 | *P* = 0.439 |  | *P* = 0.344 | *P* = 0.264 |  | *P* = 0.390 | *P* = 0.168 |

Data are HR (95% CI) unless otherwise indicated. ^*^Adjusted for energy intake and age. ^†^Adjusted for energy intake, age, gestational diabetes and gestational hypertension including preeclampsia. ^‡^Adjusted for energy intake, age, gestational diabetes, gestational hypertension including preeclampsia, maternal education, pre-pregnancy smoking and dietary fibre. ^§^Total seafood includes lean and fatty fish (salt and freshwater fish, fish-based spread), liver, roe and shellfish. ^||^Total meat includes red meat (beef, pork, mutton, processed red meat), game and poultry. ^¶^Other meat includes game and poultry.

**Table S10.** Baseline and post-intervention characteristics of the mice.

|  | Reference | Mixed diet | Total seafood | Lean fish | Fatty fish | Shellfish | Total meat | Red meat |
| --- | --- | --- | --- | --- | --- | --- | --- | --- |
|  |  |  |  |  |  |  |  |  |
| Body mass (g) |  |  |  |  |  |  |  |  |
| Start | 18.8 ± 1.2 | 19.0 ± 1.1 | 18.9 ± 1.0 | 18.9 ± 1.0 | 18.9 ± 1.0 | 18.9 ± 1.0 | 18.9 ± 0.9 | 18.9 ± 1.0 |
| Week 10 | 22.0 ± 1.5 | 21.5 ± 1.9 | 21.5 ± 1.3 | 21.8 ± 1.4 | 21.8 ± 1.7 | 21.9 ± 1.6 | 21.2 ± 1.3 | 20.9 ± 1.0 |
|  |  |  |  |  |  |  |  |  |
| Lean mass (g) |  |  |  |  |  |  |  |  |
| Start | 15.3 ± 0.9 | 15.3 ± 0.9 | 15.4 ± 0.9 | 15.5 ± 0.9 | 15.6 ± 0.8 | 15.5 ± 0.7 | 15.5 ± 0.7 | 15.5 ± 0.9 |
| Week 10 | 18.0 ± 0.7 | 17.7 ± 0.9 | 17.4 ± 1.1 | 17.7 ± 0.8 | 17.6 ± 0.7 | 17.9 ± 0.6 | 17.5 ± 0.7 | 17.5 ± 0.3 |
|  |  |  |  |  |  |  |  |  |
| Fat mass (g) |  |  |  |  |  |  |  |  |
| Start | 2.9 ± 0.7 | 2.8 ± 0.5 | 2.8 ± 0.3 | 2.8 ± 0.3 | 2.8 ± 0.4 | 2.7 ± 0.2 | 2.8 ± 0.2 | 2.8 ± 0.3 |
| Week 10 | 3.4 ± 0.9 | 3.1 ± 0.7 | 3.3 ± 0.6 | 3.3 ± 0.6 | 3.2 ± 0.5 | 3.4 ± 0.7 | 3.3 ± 0.7 | 3.1 ± 0.6 |

Data are presented as mean ± SEM.

**Supplementary Figures**

Excluded (*n* = 32,237)

- Delivery prior to 2004 or multiple particip. (*n* = 23,869)
- Missing pregnancy questionnaires (*n* = 8,368)

Source population: women recruited to MoBa in years 1999-2008

(*n* = 94,784)

Data available from the baseline questionnaire and the FFQ **Eligible for inclusion in the study (*n* = 62,547)**

**Eligible for inclusion in the analyses  *n* = 60,777**

Excluded (*n* = 1,770)

- Incomplete dietary information or invalid energy intake (<1,070 or >4,400 kcal/d) (*n* = 1,351)
- Nonviable births (<500 g or <22 weeks) (*n* = 15)
- Diabetes mellitus before pregnancy (*n* = 439)

**Figure S1.** Flow chart showing selection of the participants from The Norwegian Mother, Father and Child Cohort Study (MoBa) of women who delivered between 2004 and 2009.


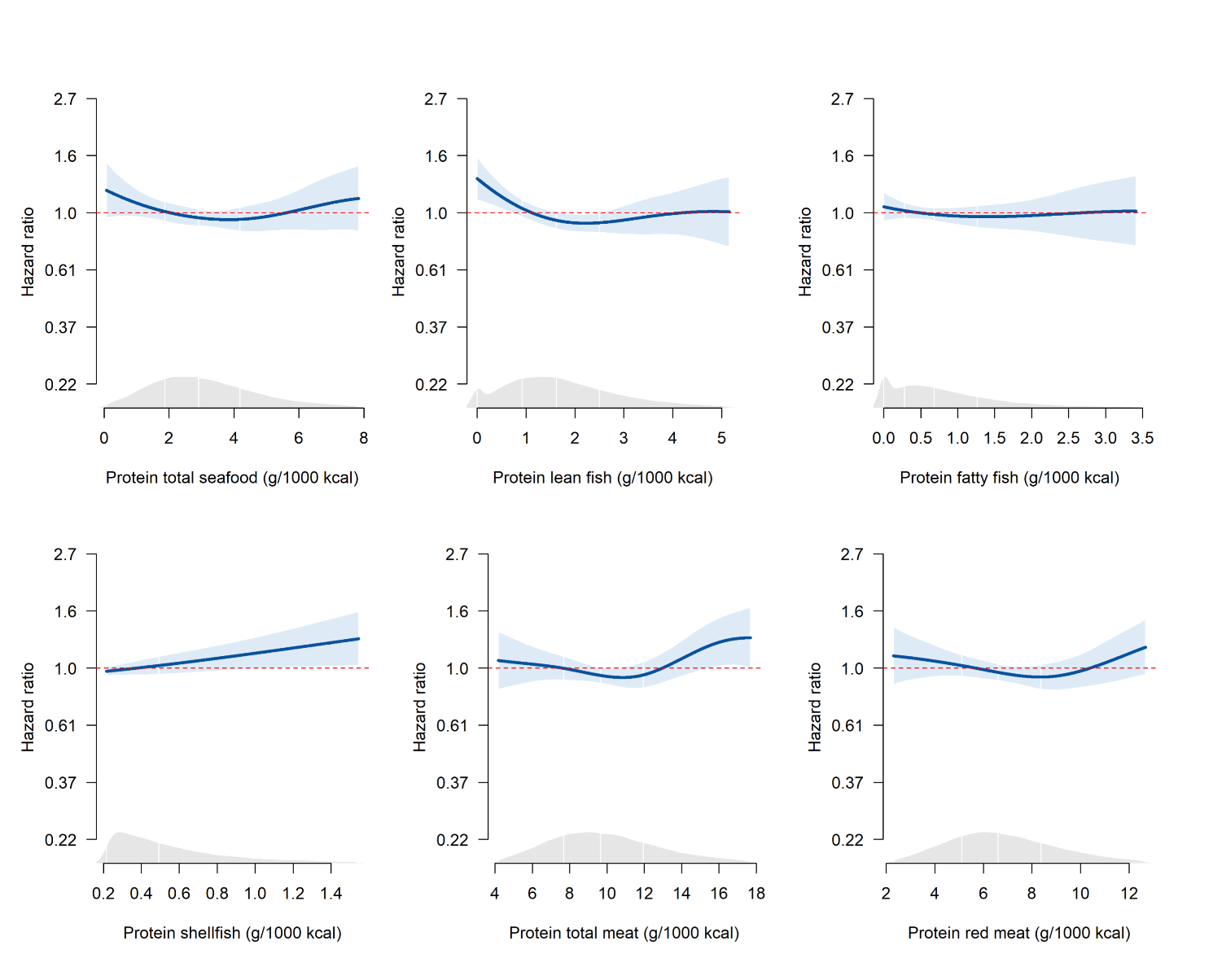


**Figure S2.** The continuous association between energy-adjusted protein intake from seafood and meat and incident type 2 diabetes (T2D) in 60,777 women with 681 T2D events. Adjusted for model 3: energy intake, age, pre-pregnancy BMI, gestational diabetes mellitus, gestational hypertension including preeclampsia, maternal education, pre-pregnancy smoking and dietary fibre. The solid lines show the observed association, the shaded areas the 95% CIs, and the density plots indicate the distribution of the protein sources. The plots are cropped at the 2.5th and 97.5th percentiles of the different protein sources. Total seafood includes lean and fatty fish (salt and freshwater fish, fish-based spread), liver, roe and shellfish. Total meat includes red meat (beef, pork, mutton, processed red meat), game and poultry.

**Figure S3.** Forest plot showing the associations (HRs and 95% CIs) between quintiles of energy-adjusted protein intake from seafood and meat (g/1000 kcal) and incident type 2 diabetes (T2D) in 60,777 women with 681 T2D events. Adjusted for model 3: energy intake, age, pre-pregnancy BMI, gestational diabetes, gestational hypertension including preeclampsia, maternal education, pre-pregnancy smoking, and dietary fibre. Total seafood includes lean and fatty fish (salt and freshwater fish, fish-based spread), liver, roe and shellfish. Total meat includes red meat (beef, pork, mutton, processed red meat), game and poultry. Other meat includes game and poultry.

**Figure S4.** (a) Body weight development (*n* = 9-10) and (b) total energy intake during 12 weeks of feeding mice a low fat reference diet and different experimental western diets based on protein from several sources (mixed), total seafood, lean fish, fatty fish, shellfish, total meat or red meat. The dotted lines represent the mean value of mice fed a low fat reference diet and a western diet based on protein from several sources (mixed). Data are presented as mean ± standard error of the mean (SEM) and different letters denote significant differences (*P* < 0.05) by one-way ANOVA using uncorrected Fisher’s Least Significant Difference (LSD) multiple comparison.


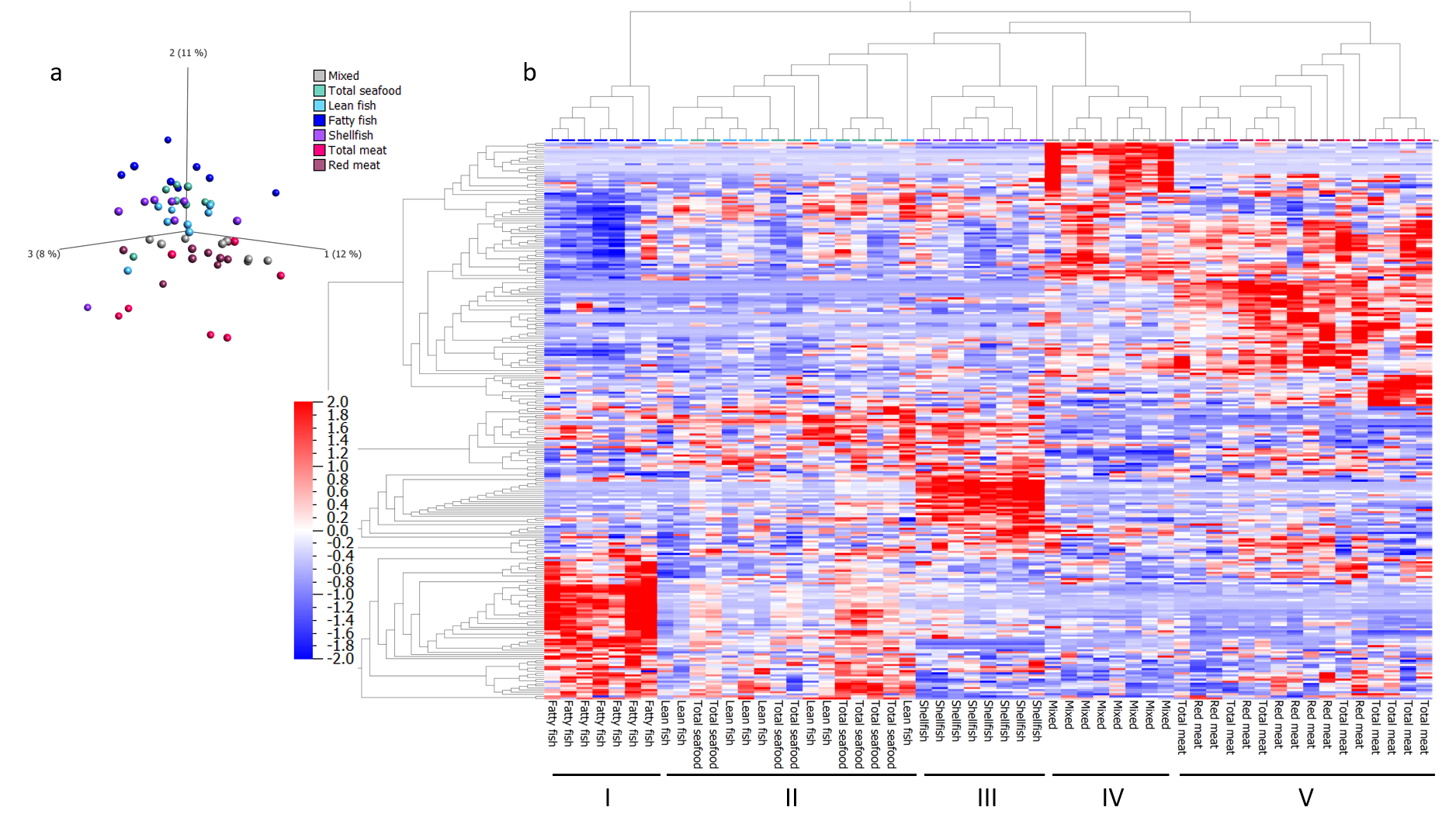


**Figure S5.** (a) Principal component analysis (PCA) of global plasma metabolomics (HD4 platform), including all 760 identified metabolites from mice fed different experimental western diets (*n* = 8) based on protein from several sources (mixed), total seafood, lean fish, fatty fish, shellfish, total meat or red meat for 12 weeks. (b) Hierarchical clustering analysis (HCA) of plasma metabolites significantly altered (ANOVA, q <0.05, 266 metabolites) between the groups fed different western diets. Separate clusters are demonstrated below by roman numbers. The color scale of the heat map indicates a change from the mean in the normalized data (mean = 0, variance = 1). Graphs were made and statistical analyses performed using the Qlucore omics explorer. Overview of all metabolites can be found in **Tables S12-13.**


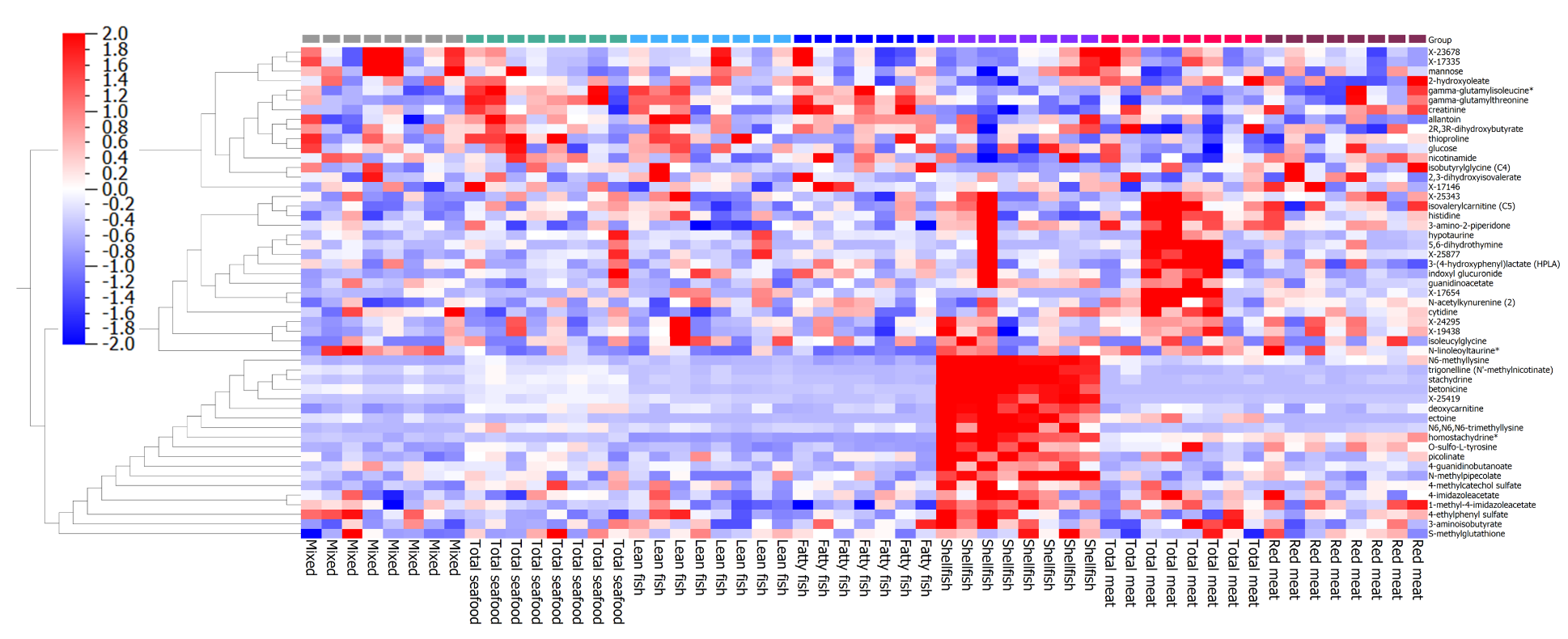


**Figure S6.** Hierarchical clustering analysis (HCA) of all plasma metabolites significantly correlated with glucose tolerance (iAUC of the GTT curve) from the global metabolomics platform (rank regression, *P* <0.05, 51 metabolites) from mice fed different experimental western diets (*n* = 8) based on protein from several sources (mixed), seafood, lean fish, fatty fish, shellfish, meat or red meat for 12 weeks. The color scale of the heat map indicates a change from mean in the normalized data (mean = 0, variance = 1). Graphs and statistical analyses were performed using the Qlucore omics explorer. Overview of all metabolites can be found in **Table S14.**


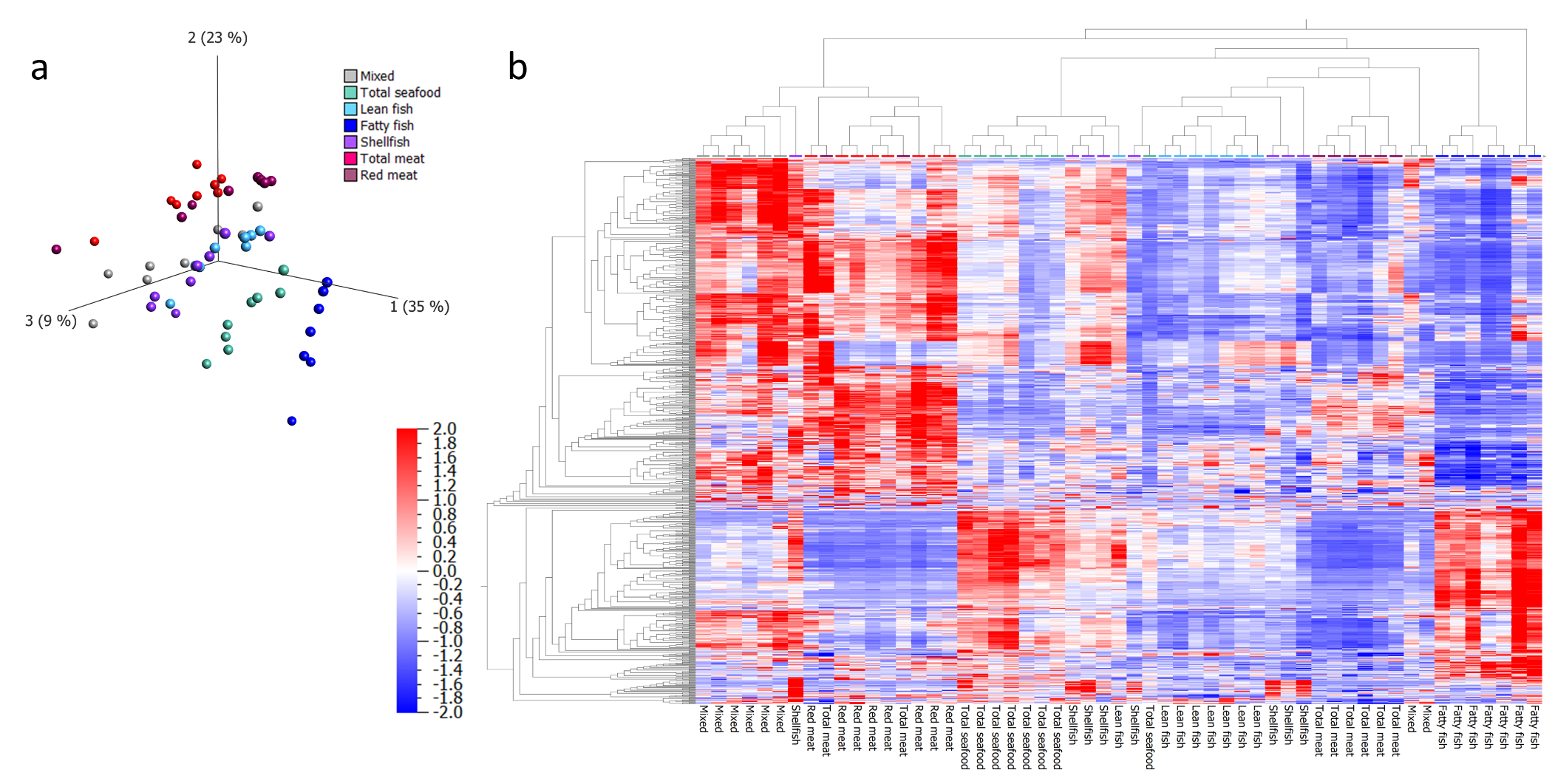


**Figure S7.** (a) Principal component analysis (PCA) of plasma metabolites measured by the complex lipid panel platform, including all 940 identified metabolites from mice fed different experimental western diets based on protein from several sources (mixed), total seafood, lean fish, fatty fish, shellfish, total meat or red meat for 12 weeks (*n* = 8). (b) Hierarchical clustering analysis (HCA) of the plasma metabolites significantly altered (ANOVA, q <0.05, 773 metabolites) between the groups fed different experimental western diets. Color scale of the heat map indicates change from mean in the normalized data (mean = 0, variance = 1). Graphs were made and statistical analyses performed using the Qlucore omics explorer. Overview of all metabolites can be found in **Tables S15-16.**


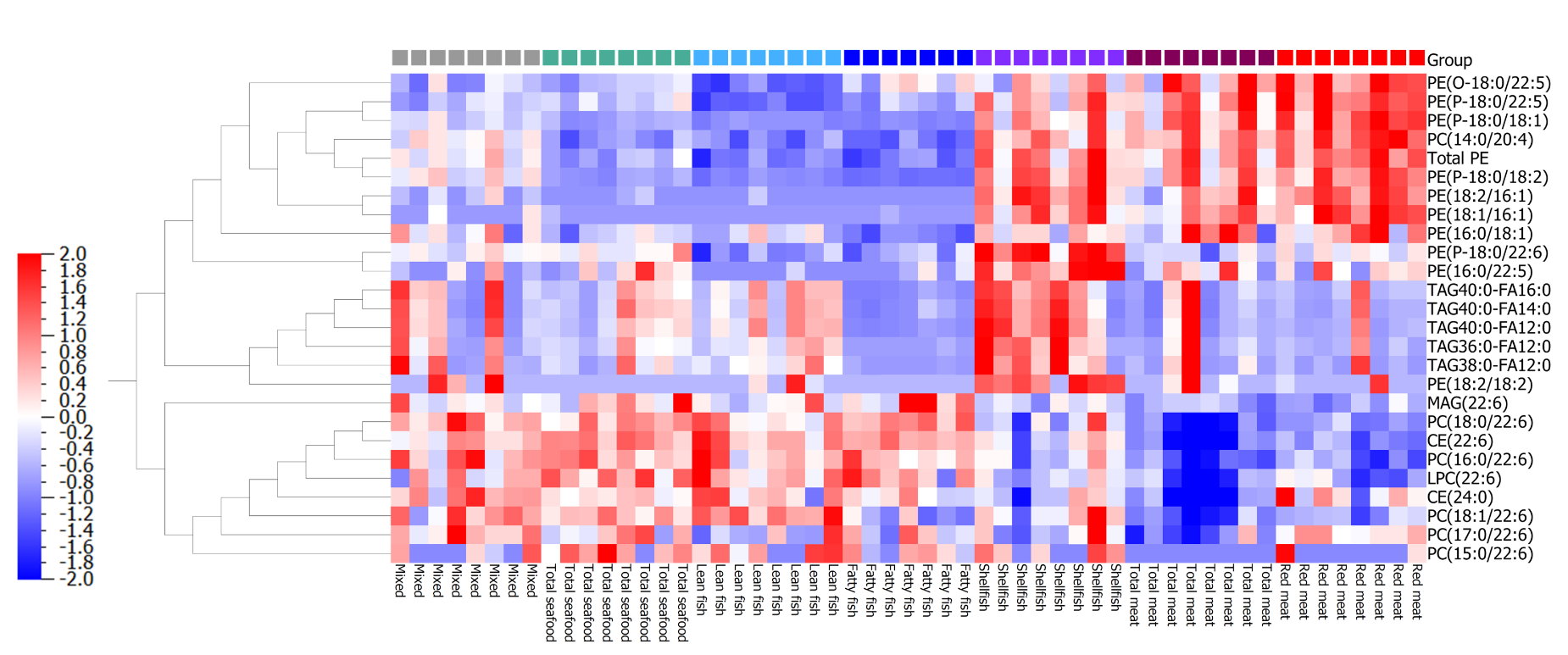


**Figure S8.** Hierarchical clustering analysis (HCA) of plasma metabolites significantly correlated with reduced glucose tolerance (iAUC of the GTT curve) from the complex lipid panel (rank regression, *P* <0.05, 26 metabolites) from mice fed different experimental western diets (*n* = 8) based on protein from several sources (mixed), total seafood, lean fish, fatty fish, shellfish, total meat or red meat for 12 weeks. Color scale of the heat map indicates change from mean in the normalized data (mean = 0, variance = 1). Graphs and statistical analyses were performed using the Qlucore omics explorer. Overview of all metabolites can be found in **Table S17**.

**Figure S9.** Illustrates the (a) correlation coefficient and (b) fold change of all the known metabolites significantly correlating with reduced glucose tolerance (iAUC of the GTT curve) from the complex lipid panel (CLP) platform (rank regression, *P* <0.05, 26 metabolites) from mice fed different experimental western diets (*n* = 7-8) based on protein from several sources (mixed), total seafood, lean fish, fatty fish, shellfish, total meat or red meat for 12 weeks. Color scale in (a) indicates the correlation coefficient for each metabolite. (b) The fold change between shellfish and the other western diet-fed groups are demonstrated for all the positive (red) and negative (blue) correlated metabolites, grouped into pathway. A list of all metabolites from the complex lipid panel (CLP) platform significantly different in the shellfish fed group compared to all the mice fed the different experimental western diets can be found in **Table S19.**


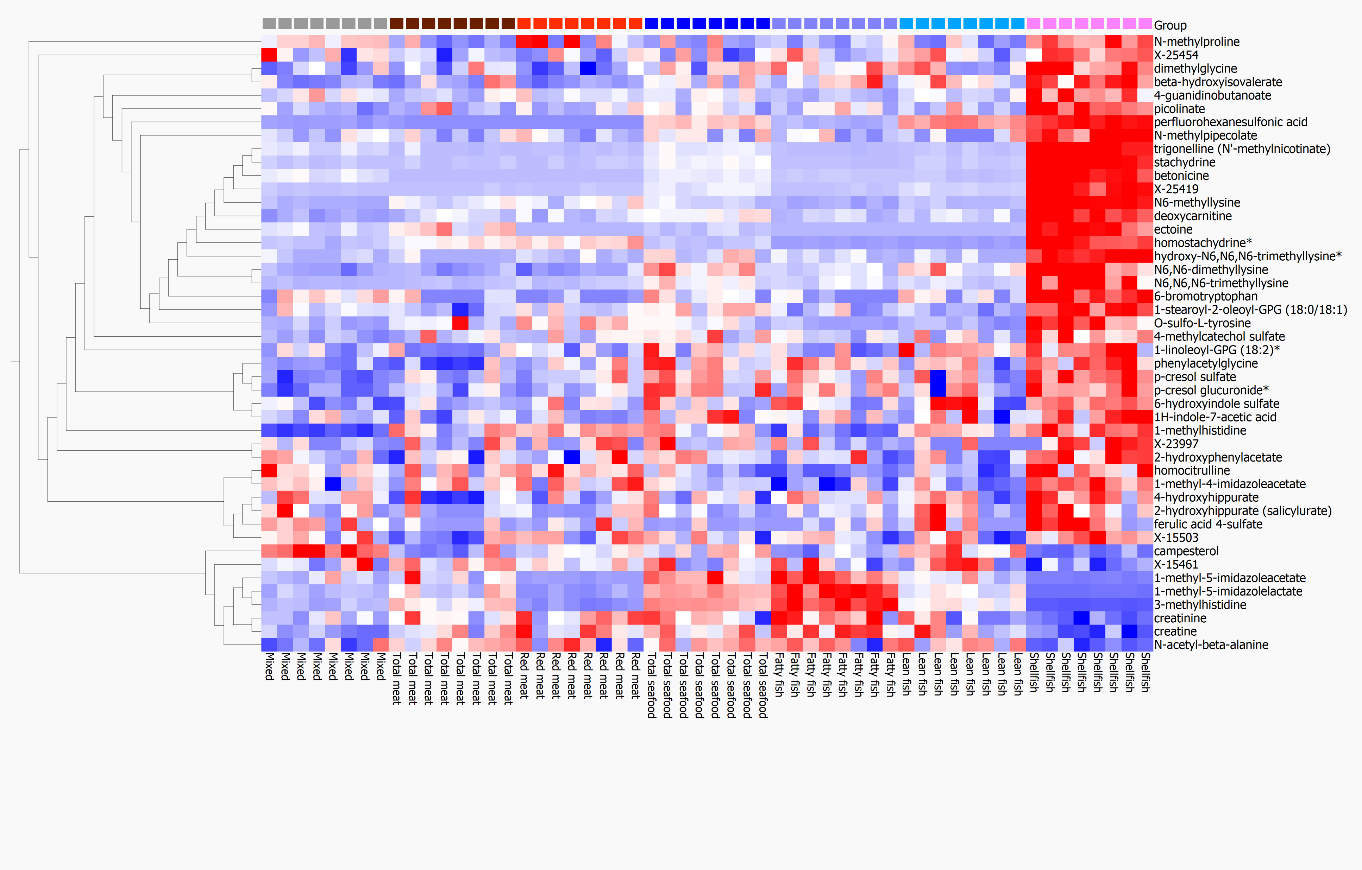


**Figure S10.** Hierarchical clustering analysis (HCA) of plasma metabolites from the global metabolomics platform (HD4) significantly different in shellfish fed mice compared to all the mice fed the different experimental western diets (*n* = 8) based on protein from several sources (mixed), total seafood, lean fish, fatty fish, total meat or red meat for 12 weeks (two group comparison, q <0.05, 41 metabolites) from mice fed different diets. Color scale of the heat map indicates change from mean in the normalized data (mean = 0, variance = 1). Graphs and statistical analyses were performed using the Qlucore omics explorer. Overview of all the regulated metabolites (known and unknown) can be found in **Table S18.**
